# Archaeological evidence of an ethnographically documented Australian Aboriginal ritual dated to the last ice age

**DOI:** 10.1038/s41562-024-01912-w

**Published:** 2024-07-01

**Authors:** Bruno David, Russell Mullett, Nathan Wright, Birgitta Stephenson, Jeremy Ash, Joanna Fresløv, GunaiKurnai Land and Waters Aboriginal Corporation, Jean-Jacques Delannoy, Matthew C. McDowell, Jerome Mialanes, Fiona Petchey, Lee J. Arnold, Ashleigh J. Rogers, Joe Crouch, Helen Green, Chris Urwin, Carney D. Matheson

**Affiliations:** 1 Monash Indigenous Studies Centre, Monash University, Clayton, Victoria Australia; 2 ARC Centre of Excellence for Australian Biodiversity and Heritage, Monash University, Clayton, Victoria Australia; 3 GunaiKurnai Land and Waters Aboriginal Corporation, Kalimna West, Victoria Australia; 4 Department of Archaeology, Classics and History, University of New England, Armidale, New South Wales Australia; 5 Everick Foundation, Brisbane City, Queensland Australia; 6 In the Groove Analysis Pty Ltd, Main Beach, Queensland Australia; 7 Laboratoire EDYTEM, Université Savoie Mont Blanc, Le Bourget du Lac Cedex, France; 8 Radiocarbon Dating Laboratory, University of Waikato, Hamilton, New Zealand; 9 ARC Centre of Excellence for Australian Biodiversity and Heritage, James Cook University, Cairns, Queensland Australia; 10 School of Physics, Chemistry and Earth Sciences, Environment Institute, Institute for Photonics and Advanced Sensing, University of Adelaide, Adelaide, South Australia Australia; 11 School of Earth Sciences, University of Melbourne, Parkville, Victoria Australia; 12 Australian Research Centre for Human Evolution, School of Environment and Science, Griffith University, Nathan, Queensland Australia

**Keywords:** Archaeology, Archaeology

## Abstract

In societies without writing, ethnographically known rituals have rarely been tracked back archaeologically more than a few hundred years. At the invitation of GunaiKurnai Aboriginal Elders, we undertook archaeological excavations at Cloggs Cave in the foothills of the Australian Alps. In GunaiKurnai Country, caves were not used as residential places during the early colonial period (mid-nineteenth century CE), but as secluded retreats for the performance of rituals by Aboriginal medicine men and women known as ‘mulla-mullung’, as documented by ethnographers. Here we report the discovery of buried 11,000- and 12,000-year-old miniature fireplaces with protruding trimmed wooden artefacts made of *Casuarina* wood smeared with animal or human fat, matching the configuration and contents of GunaiKurnai ritual installations described in nineteenth-century ethnography. These findings represent 500 generations of cultural transmission of an ethnographically documented ritual practice that dates back to the end of the last ice age and that contains Australia’s oldest known wooden artefacts.

## Abstract (web summary)

Australian archaeological finds of 12,000-year-old fireplaces and artefacts match nineteenth-century GunaiKurnai ritual practices. They represent approximately 500 generations of cultural transmission of this ritual.

## Main

Determining the longevity of oral traditions and ‘intangible heritage’ has important implications for understanding information exchange through social networks down the generations^1^. This can be achieved by tracking the origins and transmission of ethnographically known cultural practices through their associated material culture. However, understanding the issue of transmission has been fraught with difficulties. People often re-interpret and re-inscribe what they observe with new knowledge, altering the original information along the way (the hermeneutic process)^2^. Additionally, exposed material evidence can be seen for generations after a site’s construction, leaving it open to copying and re-interpretation under changing cultural contexts^3–7^. One way out of this dilemma is to discover archaeological materials that could not have later been seen and copied, but that rather needed to have been passed on through intentional information exchange, such as through formal or familial education and training^8^. In this Article, we report two examples of one such set of cultural materials from GunaiKurnai Aboriginal Country in southeastern Australia. Each consists of a wooden stick made from a *Casuarina* sp. tree stem. Each stick had been trimmed by cutting or scraping off smaller twigs flush with the stem. Each trimmed stick was smeared with fatty tissue. It was then placed in a low-temperature miniature fire, which burnt for a very short duration of time. The two installations were made deep in a secluded cave that was never used for everyday occupational activities. In each case, the miniature fireplace and its trimmed wooden artefact was rapidly buried by accumulating sediments at the Pleistocene–Holocene transition and remained in situ until they were archaeologically excavated in 2020 CE, preserving the installation’s structural integrity in the process. Such wooden artefacts and their fireplace installations were previously only known from local nineteenth-century ethnography, but have now been archaeologically found dating back to the end of the last ice age, as reported here.

The examples we document here are testimony to the endurance of cultural practices and oral traditions unaffected by complications of visibility and copying. According to nineteenth-century GunaiKurnai ethnography^9,10^, the ritual practices involving the construction of such installations took place in secluded locations. Additionally, their key wooden components normally decayed within a few years or decades, preventing them from being regularly seen by the broader population and copied over extended periods. Furthermore, the archaeological wooden objects were juxtaposed to or smeared with fatty tissue from animals or humans when they were used, matching ethnographic practice. This association of the artefacts with fat would have remained invisible to the naked eye and is thus not amenable to copying. The suite of factors contributing to the survival of both the installations and their wooden artefacts provides unparalleled insight into the resilience of GunaiKurnai narrative traditions and the passing down of knowledge. These artefacts, along with ethnographic evidence, demonstrate the transmission of ideas and practice over a timespan of 12,000 years.

The excavation methods used in this study are reported in Methods. All stages of the research comply with all relevant ethical regulations including the Australian Archaeological Association and the Australian Institute of Aboriginal and Torres Strait Islander Studies Codes of Ethics. This research was requested and led by, and undertaken with the participation of, the GunaiKurnai Land and Waters Aboriginal Corporation, representing the Aboriginal Traditional Owners of the study site. At the corporation’s request, the ethical protocols for this partnership research were formally written into a memorandum of understanding checked for ethical compliance and co-signed by the GunaiKurnai Land and Waters Aboriginal Corporation and Monash University on 23 October 2018.

## Results

### Cloggs Cave

**Fig. 1:**
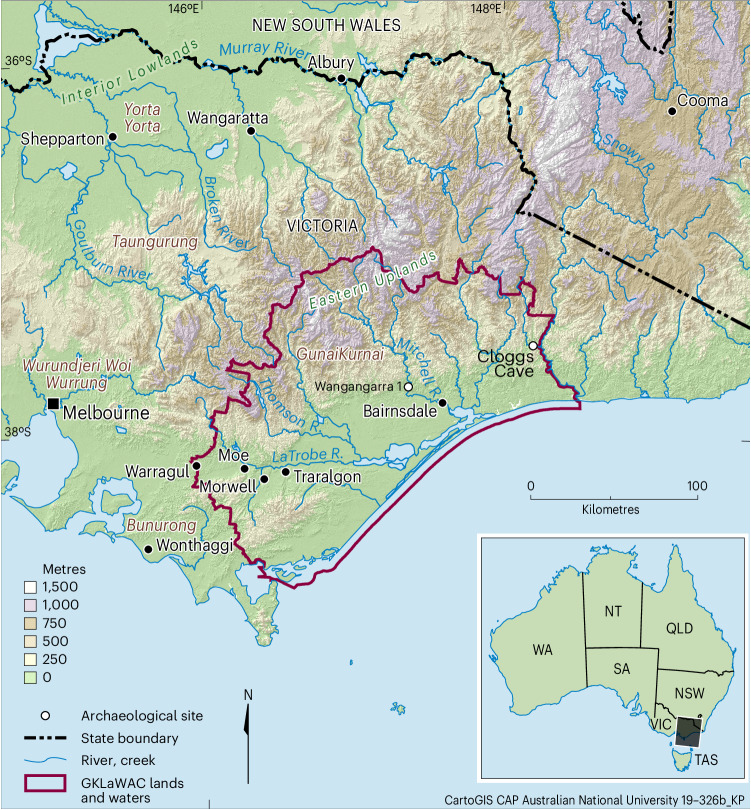
GunaiKurnai Registered Aboriginal Party area in southeastern Australia, showing the location of Cloggs Cave. The ritual installations reported in this article were excavated at Cloggs Cave.

Cloggs Cave is a 12-m-deep × 7-m-wide × 5-m-high domed cavity in outcropping limestone near the junction of the Buchan and Snowy rivers in temperate eastern Victoria, southeastern Australia (Fig. 1 and Supplementary Fig. 1)^11^. Environmental conditions inside the cave are dry and cool (~15 °C) year round, varying little seasonally. A 2 × 2 m archaeological excavation was undertaken near the middle of the main chamber in 1971–1972, some 2 m from the inner end of the entrance passage, revealing 2.4 m deep but sparse archaeological deposits adjacent to and overlying fine sediments with extinct megafaunal remains. The neutral to slightly alkaline fine sediments were rich in organic remains including leaves and other plant matter^12^. However, none of the plant material was originally analysed in detail, and none other than ash and charcoal was attributed to human activities. Our 2019–2020 excavations were undertaken against the cleaned southeast (new excavation squares P34 and P35) and northeast (new square R31) walls of the original, open excavation pit to obtain a detailed chronological sequence for the deposit, and to investigate a 12,000-year-old wood artefact whose extreme end became exposed during the cleaning of the square R31 wall (Supplementary Fig. 2)^13^.

Cloggs Cave contains a number of archaeological features characteristic of GunaiKurnai ritual installations and practices. The following ritual features date to various times that together span some 23,000 years, indicating that the cave has been used for a range of ritual activities over this period of time: (1) a stone arrangement occurs at the back of a shallow recess towards the rear of the cave (the alcove)^11^. (2) Up to 80 cm above the floor of this recess, on the alcove’s low ceiling within human reach, many of the stalactites were artificially broken. Uranium–thorium ages for the bases of ‘soda straws’ (stalactitic filament regrowths) growing on the broken stalactite stumps indicate they started growing between 120 ± 30 and 23,230 ± 300 years ago, signalling that the stalactites had been broken within the period of confirmed Aboriginal presence in the cave, which began by ~25,000 cal BP (calibrated radiocarbon years before 1950 CE)^11^. (3) On the floor adjacent to the stone arrangement is a large patch of powdered (crushed) calcite^11^. (4) A portable grindstone with traces of crushed calcite crystals, dated to between 1,535 and 2,084 cal BP, was excavated 8 m away near square P35 (refs. ^13,14^). (5) One hundred fifty-eight broken soda straws and crystal quartz artefacts were found in the excavations in squares P34–P35 and R31 (ref. ^15^). Nineteenth-century GunaiKurnai ethnography, along with current GunaiKurnai knowledge holders, identify these objects as bulk (pebbles) and groggin and kiin (crystals). Each of these object types was documented to hold ritual power and to have been used to perform magic and medicine^10,16^. (6) A fully buried standing stone, around 2,000 years old, was excavated in square P35 (refs. ^13,17^). (7) Despite the presence of tens of thousands of bones from small vertebrates (from natural deaths, mainly from owl roosts), there are no vertebrate animal food remains in the excavations^18^. (8) Local ethnography and current GunaiKurnai knowledge document that caves such as Cloggs Cave were never used for general occupation in GunaiKurnai Country; the lack of archaeological food remains in such caves is consistent with the ethnography. Rather, the caves were the retreats of mulla-mullung, powerful medicine men and women who practiced magic and rituals in secluded places^17^.

Here, we report on two additional buried archaeological installations dating to the end of the last ice age that match local nineteenth-century ritual structures, recently revealed through the excavation of square R31 at Cloggs Cave.

### Chronology

Square R31 is 50 × 50 cm in area and 153 cm deep, positioned against the exposed northeast wall of the 1971–1972 excavation pit. Before the 2020 excavation of square R31, a section of the northeast wall of the 1971–1972 pit was cleaned and its stratigraphy drawn (Supplementary Fig. 3). This exposed the end of a 39.5-cm-long wooden stick. The stick’s end was frayed, and a 1-mm-wide filament weighing 1.16 g was snipped off for accelerator mass spectrometry (AMS) radiocarbon dating. The AMS determination gave an age of 10,361 ± 30 BP (Wk-50278), equating to a calibrated age range of 11,930–12,440 cal BP at 95.4% probability (median age of 12,090 cal BP) using the SHCal20 calibration curve in OxCal 4.4. The result led to the excavation of square R31 to determine the nature of this well-preserved wooden item in the stratified deposits. Square R31 was excavated in arbitrary excavation units (XUs) averaging 2.3 cm thick, within stratigraphic units (SUs) (Methods).

A suite of 69 AMS radiocarbon ages was obtained on individual pieces of charcoal (*n* = 29), common brushtail possum (*Trichosurus vulpecula*, a herbivore) scats (*n* = 33), unburnt wood (*n* = 5) and unburnt bark (*n* = 2) from square R31 (Supplementary Table 1 and Supplementary Fig. 3), along with ten single-grain optically stimulated luminescence (OSL) ages from across the nearby stratigraphy exposed by the 1971–1972 pit^19^.

The 57 AMS radiocarbon ages obtained across the different dated materials from XU43 (in the SU4U–SU4V interface) to XU1 (SU2A) show excellent chronostratigraphic correspondence, with minimal age reversals. The findings presented here all came from this part of the stratigraphy. Bayesian modelling of all age determinations from the northeast wall of the 1971–1972 excavation pit (69 AMS radiocarbon and 2 OSL ages) indicates that XU43 to XU1 spans the period from 25,740 to 1,460 cal BP^19,20^. The Bayesian-modelled ages are indicated and rounded to the nearest 10 years by convention, to differentiate them from individual unmodelled calibrated ages (Supplementary Table 2).

### The excavated ritual installations

**Fig. 2:**
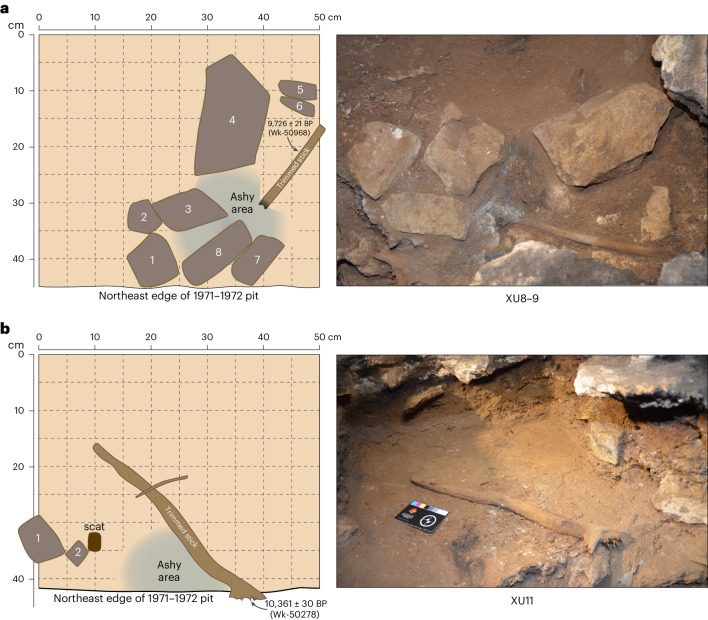
The two miniature fireplaces with trimmed sticks immediately after they were exposed by excavation in Cloggs Cave square R31, with the sticks’ bases not yet separated from the sediments in which they sit. **a**, The installation from XU8–9 (SU4D) dates to 10,720–12,420 cal BP (Bayesian-modelled age). **b**, The installation from XU11 (SU4E) dates to 11,420–12,950 cal BP (Bayesian-modelled age).

The two ritual installations from square R31 were archaeologically excavated in XU11 (in SU4E) and XU8–9 (in SU4D). Each consists of a miniature fireplace with an emanating wooden stick. The one from XU11 lay at an average depth of 43.2–47.5 cm below ground. The Bayesian model dates it to 11,420–12,950 cal BP (youngest and oldest possible ages from two boundaries at 95% probability), consistent with the direct AMS radiocarbon age of 10,361 ± 30 BP (Wk-50278), individually calibrated to 11,930–12,440 cal BP, obtained from the stick itself (see above). The fireplace consists of an ashy deposit 4.3 cm thick. The trimmed wooden stick lies on its side, with the outer edge of the sparsely spread ash from the fireplace abutting it (Fig. 2b).

**Fig. 3:**
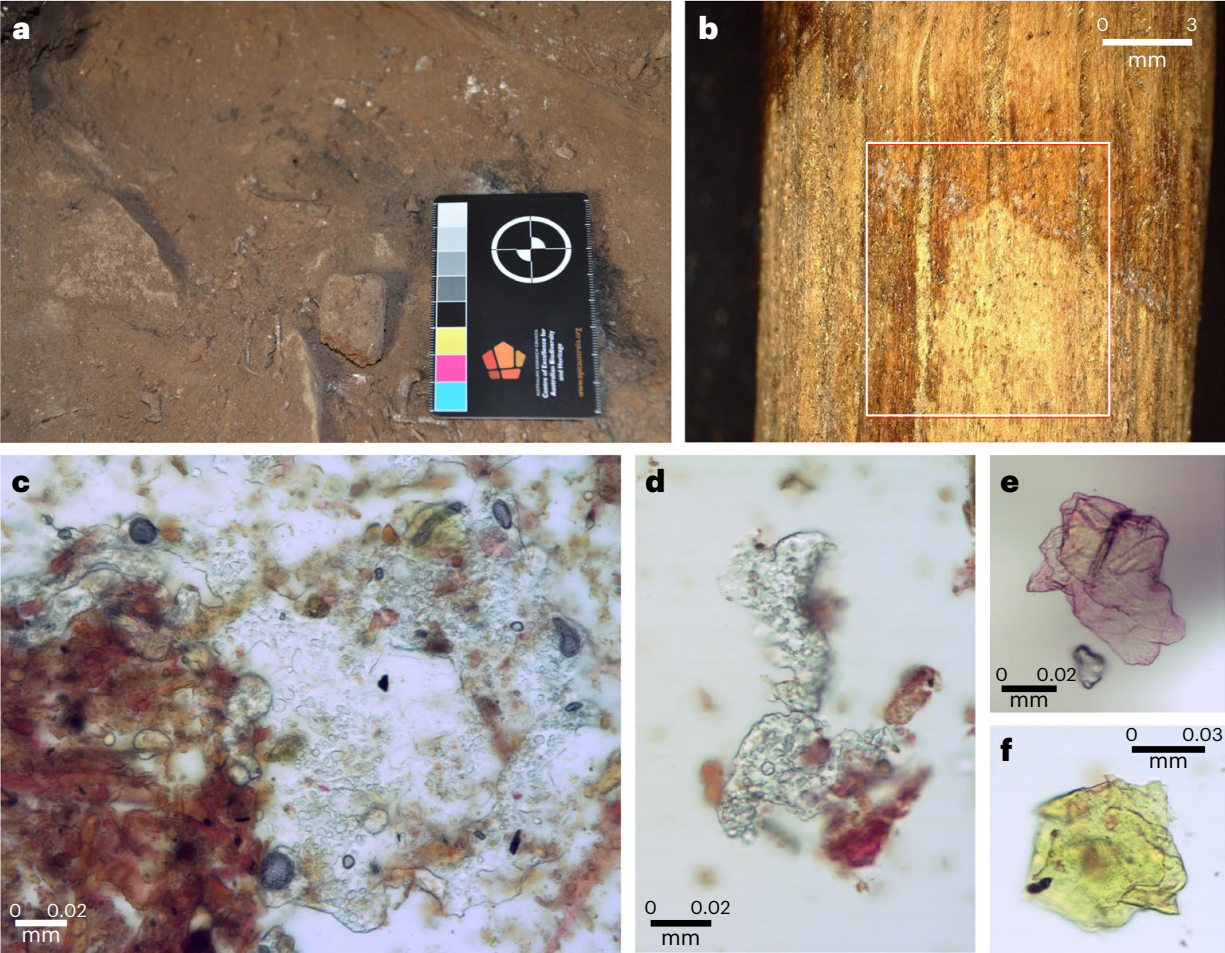
Soft tissue on wood artefacts from Cloggs Cave square R31. **a**, *V.* *ursinus* scat manually positioned on the installation from XU11. **b**, The location of lipid extraction (shown in **c** and **d**) on the trimmed stick from XU8–9. Note the palaeo-staining of the wood; the lipids came from the lighter section of the wood in the lower right quarter of the photograph (photographed at ×30 magnification under cross-polarized light). **c**,**d**, Two examples of lipid residues (clear transparent ‘bubbly’ film) on the trimmed stick from XU8–9 (photographed at ×400 magnification under part-polarized light). **e**,**f**, Lipid or keratin-like fragments on the 11,420–12,950 cal BP (Bayesian-modelled age) trimmed stick from XU11. In **e**, it has taken up the PSR stain and is thus stained pink, while the stain in **f** is more diffuse and has only been taken up along its peaks and ridges, as represented by the thin pink lines in the fragment (photographed at ×400 magnification under part-polarized light).

A single scat was discovered on the edge of the fireplace (Figs. 2b and 3a). Its size, shape and content correspond to typical scats from the common wombat (*Vombatus ursinus*), the only wombat species known to have lived in southeastern Australia during the terminal Pleistocene and Holocene. *V.* *ursinus* is a fossorial graminivore (feeding principally on grass) that defecates multiple scats at once^21^. It does not normally occupy caves. The scat is whole and unburnt and is the only wombat scat in the entire deposit. Its location immediately on the edge of the fireplace indicates that it was manually placed there as part of the fireplace-with-stick installation during its use between 11,420 and 12,950 cal BP (Bayesian-modelled age).

The uppermost and slightly younger installation came from XU8–9 in SU4D, its centre 12 cm east and stratigraphically 4 cm above the lower installation (Fig. 2a). The installation has a Bayesian-modelled age of 10,720–12,420 cal BP. This is consistent with an AMS radiocarbon age of 9,726 ± 21 BP (Wk-50968) on a small outer filament of the XU8–9 stick from the installation, which individually calibrates to 10,870–11,210 cal BP. The installation retained a very high level of structural integrity at the time of excavation, signalling an archaeological feature that was rapidly buried and remained preserved much as it was left when last used. The fireplace consists of eight limestone rocks enclosing an irregular burnt area of maximum 15–20 cm diameter containing only 22 tiny pieces of charcoal (totalling 0.29 g, as retained by the 2-mm-mesh sieve) and rich in ash. Each rock is lightly burnt and weighs between 159.7 and 2,106.4 g. The ashy deposit lies 39.4–42.5 cm below ground and is 3.1 cm thick. A small quartz flake and volcanic manuport from XU8 were found near the XU8–9 installation (stone artefacts #3 and #2, respectively, of Table 9 in ref. ^15^).

**Fig. 4:**
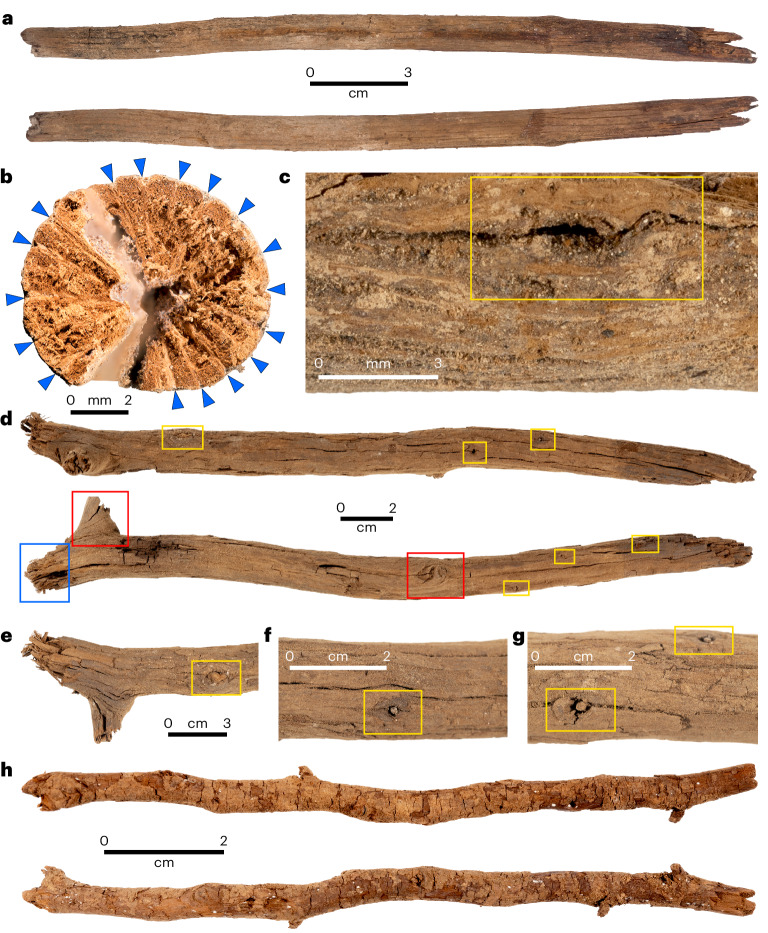
The three largest pieces of wood from the two miniature fireplaces at Cloggs Cave, showing details of the two trimmed sticks. **a**, The trimmed stick from the XU8–9 fireplace. **b**, End view of the XU8–9 trimmed stick, showing the large rays (blue arrows) characteristic of *Casuarina* spp. At this magnification, the smaller rays are not visible. The number of large rays is characteristic of *C.* *cunninghamiana*. The vessel distribution and vessel sizes are relatively uniform. **c**, The proximal end of the trimmed stick from the XU8–9 fireplace. The remnant base of a twig trimmed off flush with the smooth surface of the stick is evident (yellow rectangle). **d**, The trimmed hooked stick from the XU11 fireplace. The distal end is charred. The blue rectangle shows the proximal end from which the torn and fibrous end was broken from the tree. The red rectangles show larger twigs that were trimmed or broken off. The yellow rectangles show small twig junctions cut or scraped off flush with the stem, creating a smooth shaft on the stick. **e**, Torn fibrous proximal hooked end of the trimmed stick from the XU11 fireplace. The fibrous elements indicate that the wood was green when broken. The base of a small twig cut or scraped flush with the main stem is evident (yellow rectangle). **f**,**g**, Three different bases of twigs (yellow rectangles) cut or scraped flush with the main stem of the stick from the XU11 fireplace. **h**, Small twig from the XU11 fireplace. It exhibits no signs of twig removal nor charring (photos by Steve Morton).

The two trimmed wooden sticks associated with the XU11 and XU8–9 fireplaces each have a lightly charred tip (Fig. 4a,d), the one from XU8–9 still emanating from the centre of its ashy area. The latter stick lay flat on the ground when excavated and appears to have been left in the position it was last used, as indicated by its burnt tip that merges undisturbed with its corresponding ashy sediment in the fireplace (Fig. 2a).

### The wooden artefacts

The two wooden artefacts from the XU11 and XU8–9 installations are both of *Casuarina cunninghamiana* or *Casuarina glauca*; the two species cannot be readily distinguished through their wood anatomy. The diagnostic anatomy of both wooden artefacts is detailed in ‘Identification of the wood’ in Methods, Supplementary Table 3 and Supplementary Fig. 4a,b. Typically, branches and stem wood form compression and tension wood for strength and to resist gravity, causing growth to be non-uniform around the core. In contrast, the relatively uniform cell structure around the core of the two Cloggs Cave sticks suggests they originated from wood that grew upright.

The 11,420–12,950 cal BP (Bayesian-modelled age) wooden stick from XU11 is 39.5 cm long and 1.3–2.5 cm wide along the stem, widening to 4.3 cm at its proximal end as it forks into its juncture with either another branch or trunk stem (Fig. 4d,e). ‘Proximal’ refers to the wood closest to the heartwood of the tree and ‘distal’ the furthest. The stick is very smooth along its entire length, except where it forks at the proximal end where some bark is retained. The fraying of the proximal end of the stick with long fibres (Fig. 4e) signals that it was manually broken while green (living or recently living). The distal end of the stick has some darkening, possibly from burial or from surface adhesion (fats), but most probably from slight burning (Fig. 4d). Heat exposure appears to have been fleeting, as full charcoalification has not occurred. Charring extends just 1–2 mm below the surface and does not show signs of prolonged heat exposure. The area subjected to direct heat is approximately 5 cm from the distal end (archaeologically this was the end closest to the fire). A small area circa 3 cm from the proximal end (archaeologically the end distal to the fire) also displays signs of fleeting exposure to heat. The stick has been deliberately altered: five smaller twigs have been trimmed off the stem (Fig. 4d–g), cut or scraped flush with the stick’s smooth surface. As the areas where the smaller twigs were trimmed off are not burnt, it is not possible to tell whether the trimming occurred before or after the distal end of the stick was burnt. On the basis of its morphology and burning condition alone, the stick appears to have (1) been used to stoke a small fire or (2) fallen out of the fire before complete charcoalification occurred. The act of neatly trimming smaller twigs flush with the stem to make the stick’s surface smooth and straight is unusual. If the stick was used solely to start or fuel a fire, there would have been no need to remove these smaller branches. This therefore suggests a different use for the XU11 trimmed stick.

The 10,720–12,420 cal BP (Bayesian-modelled age) stick from XU8–9 is 19.7 cm long and 0.7–1.2 cm in diameter. There is charring that extends 0.2–0.3 cm subsurface at one end and for some 1.5 cm along the stem, but the rest of the stick remains uncharred. Charcoalification is again incomplete, meaning that the stick was burnt for only a short time before the fire was extinguished. We can discount its removal from a longer-burning fire, as its charred distal end remained in situ in the ashes of the fire when it was excavated (Fig. 2a). The stick is very straight and shows the same unusual trait as the XU11 one dated to 11,420–12,950 cal BP (Bayesian-modelled age), in that the XU8–9 stick had its two lateral twigs trimmed off, flush with the stick’s surface. The XU8–9 stick must have been trimmed before its placement in the fire given that it was not moved after the fire burned (Figs. 2a and 4a,c).

### Residue analyses of the XU11 and XU8–9 trimmed sticks

At no stage during excavation or analysis were the wooden artefacts touched by human hands; handling was minimal and always took place through new disposable powder-free nitrile gloves. Residue analyses of the surface of the XU11 trimmed stick shows the presence of lipid or keratin-like faunal tissue fragments, indicating that the stick had come in contact with animal or human body tissue either through intentional smearing or juxtapositioning (Fig. 3e,f and ‘Residue analysis’ in Methods). Residue analysis along the surface of the XU8–9 stick shows the presence of even more extensive patches of animal or human lipids (Fig. 3c,d). Here there is a discrete colour-differentiated area outlined by a carbonized margin. While the majority of the residue sample was extracted within the lighter area, some material from the carbonized margin was included. Lipids cover the non-carbonized lighter-coloured area of the wood, probably protecting this section of the wood in the process (Fig. 3b). Chemical analysis using both liquid and gas chromatography–mass spectrometry (LC–MS and GC–MS) identified 16 fatty acids including those found in abundance in animal or human fat (palmitic acid, palmitoleic acid, stearic acid, oleic acid, linoleic acid and linolenic acid) and fatty acids found only in some animal species (pentadecanoic acid, myristoleic acid and margaric acid) including some native Australian animal species. In addition, there were 13 glycerides identified supporting the animal or human origin of these compounds, and seven dicarboxylic acids including four that comprise common breakdown products of the identified unsaturated fatty acids (Supplementary Table 4 and Supplementary Fig. 5). This indicates that part of the stick had been smeared with fat or fatty acids of a type associated with animal or human fat when it was used. There are no macroscopic or microscopic signs of leaves from which oils may have originated on or near the stick, and *Casuarina* trees only have lipids associated with their leaves. Given the presence of fatty acids that are exclusively present in animals or humans, plant oils could not have been the source of the lipids on the stick.

The XU11 and XU8–9 miniature fireplaces are unusual in Australian archaeology, both for their small size and for their association with a single, slightly singed but largely unburnt straight, trimmed stick. Given the superb conditions conducive to the preservation of unburnt wood, the absence of other substantial pieces of wood other than small to tiny fragments of thin twigs (see below) is noticeable. Had the two installations been used as cooking or heating fires, we would expect to find numerous other, variably burnt sticks and considerably larger-sized charcoal emanating from larger branches and logs. Similarly, if the two sticks were placed in the fire as fuel, they would have been more burnt and there would have been no need to trim them straight. Furthermore, the extensive spread of lipid residues on the XU8–9 stick, and the less-dense spread of adipose lipid or keratin-like tissue fragments on the XU11 stick, are unusual and have not previously been reported from any archaeological site in Australia. The standard heating and cooking explanations for the construction of the fires and their associated wood artefacts are insufficient and do not adequately account for the archaeological finds, neither individually nor as multi-component installations.

### Other wooden items

XU8–9 contains 64 other very small pieces of unburnt wood and one of unburnt bark. The taxa of the bark and of 34 of the pieces of wood (maximum length of 0.3 cm) are unidentified. The 30 identifiable small pieces of wood each measure between 0.8 cm and 5.5 cm maximum length and are all of *C.* *cunninghamiana* or *C.* *glauca*. In addition, one tiny piece of charcoal weighing 0.15 g and measuring 4.9 mm long × 2.2 mm wide is from *Hedycarya angustifolia* (Australian native mulberry), a species of wood widely used to make fire drills in Aboriginal Australia during ethnohistoric times^22^. Local cultural knowledge of the use of *H.* *angustifolia* to make fire drills is retained today by GunaiKurnai Elders. The other 21 fragments (totalling 0.14 g) of charcoal from the XU8–9 fire are too small to identify beyond Dicotyledon cf. Monimiaceae, which is the same family as *H.* *angustifolia*. Thus, they could all be small fragments from the same source.

XU11 features two additional pieces of wood, one 2.7 cm long, the other 11.6 cm. The shorter piece exhibits signs of burning. They are both *C.* *cunninghamiana* or *C.* *glauca*. The longer piece is a small stick and is notably different from the two main sticks discussed previously, exhibiting no evidence of trimming of smaller twigs (Fig. 4h). It could be a trimming of the larger stick found in XU11—the width of its base matches the widths of the trimmed-off twigs on the larger stick—but no conclusive connection such as matching trim marks has been identified. It was, however, found lying across the larger burnt stick of XU11. The shorter burnt piece may also be a broken fragment of the larger stick.

### Nineteenth-century GunaiKurnai ethnography

The absence of animal food remains in or near each of the two fireplaces at Cloggs Cave square R31, their small size, the presence in each of a single straight, trimmed *Casuarina* stick only fleetingly burnt at a low temperature, and each stick having come into contact with animal or human fatty tissue and, in the case of the XU8–9 stick extensively so, all indicate a peculiar design. These shared features suggest that each fireplace installation was uniquely crafted for purposes other than cooking or heating.

Nineteenth-century ethnography provides a good description of the function of such fireplaces. In 1887, Alfred Howitt, government geologist and pioneer ethnographer, published details of the ritual practices of the GunaiKurnai and neighbouring groups from southeastern Australia. He documented the activities of individuals referred to as ‘sorcerers’, ‘wizards’ or medicine men and women, who performed magic on their victims or healed the dying—what anthropologist Adolphous P. Elkin^23^ later called Aboriginal men and women ‘of High Degree’. Among the GunaiKurnai, such powerful individuals were known as ‘mulla-mullung’, an office within GunaiKurnai society acquired through ritual training^16,24^. Ritual spells cast on victims were conducted in secluded places, away from prying eyes. Howitt described how magic was employed to harm a victim using a ritual fire and a wooden object smeared or attached with a piece of human or animal fat (major sources of lipids):

“In all these tribes a general, I may say almost an universal, practice has been to procure some article belonging to the intended victim. A piece of his hair, some of his faeces, a bone picked by him and dropped, a shred of his opossum rug, or at the present time of his clothes, will suffice, or if nothing else can be got he may be watched until he is seen to spit, when his saliva is carefully picked up with a piece of wood and made use of for his destruction…

The Kurnai practice is to fasten the article [something that belonged to the victim] to the end of a throwing stick, together with some eaglehawk feathers, and some human or kangaroo fat. The throwing stick is then stuck slanting in the ground before a fire, and it is of course placed in such a position that by-and-by it falls down. The wizard has during this time been singing his charm; as it is usually expressed, he ‘sings the man’s name,’ and when the stick falls the charm is complete. The practice still exists.”^9^

The wooden instrument activating these rituals generally took the form of magical throwing sticks that mimicked spear throwers, often renowned for their power. These wooden implements were called ‘murrawan’, and their makers and users ‘bungil murrawun’^16,24^. In 1925, Presbyterian minister and amateur ethnographer John Mathew described them as follows: “At one end it had a hook like that on the wommera (woomera; spear-thrower), the lever for spear-throwing, and at the other a bunch of eaglehawk feathers”^25^. The hooked shape at the end of the stick associated with the 11,420–12,950 cal BP (Bayesian-modelled age) installation from XU11 is reminiscent of these implements (Fig. 4e). Nineteenth-century colonial collectors acquired many of these ritual objects; when one was not available, replacements charged with ritual power through song and application of bodily substances were used^9^.

Howitt also described “the belief of the Kurnai that men called ‘bunjil barn’ could cause their victims to walk to them by reason of their enchantments… the pieces of wood from which they received their name were… made of the Casuarina. Their magic fire round which they danced, singing the name of their intended victim, is exactly the magic fire (tálmarū) of the Murring initiation ceremonies and the bunjil barn being rubbed over with charcoal followed the custom of the initiation”^9^.

The closely related and visually similar genera *Casuarina* and *Allocasuarina* were particularly important trees in this regard (the two genera were not botanically differentiated until the late 1900s, that is, the ethnography only refers to ‘*Casuarina*’). Ethnographers Lorimer Fison and Alfred Howitt also made the connection between *Casuarina* and rituals involving death among the GunaiKurnai. “Not only, therefore, is death in some cases attributed to the acts of a sorcerer, who may be any man they meet, but death is also believed to occur by a combination of sorcery and violence. Such a proceeding is that known as Barn”, they wrote, specifically noting that ‘barn’ is ‘the Casuarina’^10,24^.

## Discussion

Well-preserved wooden artefacts are rarely found in Pleistocene and Early Holocene archaeological sites. In Australia, contrasting wet and dry seasons affect many sedimentary contexts, stimulating microbial activity that increase the rate of organic degradation^26^. The wooden artefacts reported here are Australia’s oldest, joining a unique array of wooden artefacts from a peat layer at Wyrie Swamp (South Australia) as the only other known wooden artefacts dating to the Pleistocene–Holocene transition^27^. The close similarities between the Cloggs Cave miniature fires and their trimmed, lipid (animal or human fat)-smeared wooden sticks and mid-nineteenth-century ethnographic descriptions, signal the continuity of ethnographically known practices from parallel antecedents dating back some 12,000 years to the end of the last ice age.

Our study needs to be understood by the limitations of archaeological evidence, which relies on material remains to infer past behavioural performances rather than directly observing the performances themselves. In this light, we have found an exceptional convergence of factors—the rare preservation of wooden items, both of *Casuarina* and juxtaposed or smeared with animal or human fatty substances; the repeated construction of near-identical miniature fireplace installations built and used circa 1,000 years apart; the extraordinary preservation of the final state of a ritual act; and GunaiKurnai ethnographies that describe ritual installations akin to those found in the archaeology. This unique combination indicates the transmission of a very specific local cultural practice over 12,000 years. These findings are not about the memory of ancestral practices, but of the passing down of knowledge in virtually unchanged form, from one generation to the next, over some 500 generations.

## Methods

In 2017, the GunaiKurnai Land and Waters Aboriginal Corporation sent a delegation to Monash University to request long-term partnership research on GunaiKurnai traditional lands and to undertake archaeological excavations at Cloggs Cave. The corporation is the registered Aboriginal Party that represents the GunaiKurnai people, the Aboriginal Traditional Owners of Cloggs Cave, as determined by the Victorian Aboriginal Heritage Council under the Aboriginal Heritage Act 2006. The GunaiKurnai Land and Waters Aboriginal Corporation was established in 2007 and on 22 October 2010, the Federal Court recognized that GunaiKurnai hold Native Title rights over much of Gippsland. On the same day, the State of Victoria entered into the first Recognition and Settlement Agreement with the GunaiKurnai under the Traditional Owner Settlement Act 2010.

The archaeological excavations presented here were undertaken to investigate GunaiKurnai history at the request and with the participation of the GunaiKurnai Land and Waters Aboriginal Corporation. All the laboratory analyses reported here were undertaken with the corporation’s fully informed approval as part of this research programme. The research was undertaken through Cultural Heritage Permit GKRAP-19-0001, issued on 14 January 2019 under the Aboriginal Heritage Act 2006. All aspects of the project adhere to the Australian Indigenous Data Sovereignty Principles, the AIATSIS Code of Ethics for Aboriginal and Torres Strait Islander Research, and the Australian Archaeological Association Code of Ethics.

By law in Victoria, Australia, Indigenous cultural materials (including archaeological materials) remain the property of the Aboriginal Traditional Owners. This is the case with all the materials reported here. The samples do not have accession numbers and will be returned to the GunaiKurnai Land and Waters Aboriginal Corporation, who will in due course decide how and where to store or display the materials. No geological or palaeontological specimens were used in our study.

### Archaeological excavation

In 2020, a 50 × 50 cm area designated ‘square R31’ was excavated against the still-exposed, cleaned northeast wall of Josephine Flood’s 1971–1972 excavation pit at Cloggs Cave. In the footprint area of square R31, the uppermost 20 cm of deposit that previously made up the surficial SU1—a stratigraphically well-defined culturally sterile sandy loam layer—had been removed by Flood during the 1971–1972 excavations, so that the top of SU2 was then exposed. In 1972, immediately after completion of her excavations, Flood covered the exposed but otherwise undisturbed SU2 surface with protective plastic sheeting, on top of which she laid a 20-cm-thick layer of introduced fine sediments. Upon removal of these fine sediments and plastic sheeting in 2020, the protected SU2 surface was found to have remained intact, as confirmed by extant conditions and comparisons with the photographs, the detailed map of the SU2 surface and written records made in 1971–1972.

The 2020 excavation of square R31 was undertaken from the SU2 surface downwards in average arbitrary XUs of mean 2.3 cm thickness following the stratigraphy. The excavated sediments were immediately bagged by XU without sieving, so that all sieving and sorting took place under controlled conditions at the Monash Indigenous Studies Centre archaeology laboratories, Monash University. In the field, in situ sedimentary ancient (seda)DNA sediment samples were also collected in conical-bottom Corning 50-ml centrifuge tubes before excavation of each XU. Upon completion of the excavation, two sets of sediment samples were collected in paired 1.5-cm-diameter, 15-ml conical-bottom plastic centrifuge test tubes at 5 cm depth intervals, one tube again for sedaDNA analysis and the other for pollen and phytolith analyses. Once these procedures were completed, five in situ block-oriented sediment samples were collected for micromorphological analysis. During the excavation and in the laboratory, no cultural materials were handled with bare hands: square R31 was archaeologically excavated and analysed with disposable powder-free nitrile gloves. All cultural materials seen during the excavation were individually double-bagged in labelled new resealable zip-lock bags, without any label or ink ever touching the artefacts. In the laboratory, circa 100 g sediment samples of unsieved sediment were separated from each XU bulk sediment bag, double-bagged in labelled new resealable zip-lock bags and kept for sediment analysis and as supplementary samples for pollen and phytolith analysis.

In the laboratory, the excavated sediments other than the sediment samples and in situ finds were then wet-sieved in 2-mm-mesh Endicott sieves and air dried for 4–7 days before manual sorting with tweezers. All cultural materials were bagged by XU. The <2 mm residue fraction from the wet sieving was air dried and, along with all the non-artefactual sediments retained (that is, the ≥2-mm-wide fraction) after sorting, were returned to Cloggs Cave, as per the appropriate GunaiKurnai cultural protocols for ancestral materials (which includes all objects and sediments from the past). All the archaeological materials (stone and wood artefacts, sediment samples, sedaDNA samples, pollen samples and micromorphology blocks) and associated animal bones (mainly from owl roosts)^18^ are currently retained in the specialist university laboratories charged with their respective analyses and will be returned to the GunaiKurnai Land and Waters Aboriginal Corporation at the completion of the research project. The two trimmed wooden sticks reported here were analysed for taxonomic identification and wood modification by N. Wright, and for microscopic residues by B. Stephenson. The lipids extracted from the wooden sticks by B. Stephenson during the microscopic residue analysis were analysed by GC–MS and LC–MS by C. Matheson. The analytical methods used are reported below. None of these laboratory analysts were aware of the local GunaiKurnai or neighbouring ethnography before their analyses and reports: the laboratory analyses and wood and residue identifications were undertaken entirely independently of the GunaiKurnai and broader regional ethnography.

The base of the square R31 stratigraphic sequence comprises culturally sterile SU5A and SU5B (120–153 cm depth)—an extinct megafauna layer—overlain by a stacked sequence of 22 sublayers of SU4 (SU4A–SU4V, 35–120 cm depth). SU4V and SU5A are intermixed and rocky at their interface (119–144 cm depth) due to ceiling rockfall. SU4 and SU5 are loam to sandy loam. SU3, an infilled sinkhole confined to an area >1 m south of square R31, is not present in the square R31 stratigraphy^11^. Above SU4 are seven stacked sublayers of SU2 (SU2G–SU2A, at 20–35 cm depth), consisting of loam to sandy loam and ashy layers from anthropogenic fires. The pHs of SU5–SU1 range between 7 and 8. SU1, the culturally sterile surficial sandy loam removed by Flood in 1971–1972, does not feature in our excavation. The square R31 stratigraphy and radiocarbon ages are shown in Supplementary Fig. 3.

Sixty-nine AMS radiocarbon ages were obtained from square R31 (Supplementary Table 1), along with ten single-grain OSL ages from the nearby stratigraphy exposed by the 1971–1972 pit. Another 40 AMS radiocarbon ages were obtained from squares P34–P35 located 2–2.5 m from square R31, enabling the R31 sequence to be positioned in the cave’s broader chronostratigraphy^13^. The AMS radiocarbon determinations from square R31 XU44–XU50, representing the deepest XUs covering SU5 up to the SU4U–SU4V interface, have minor chronostratigraphic reversals signalling mixing from roof fall. The 57 AMS radiocarbon ages from the higher stratigraphic levels are all in excellent chronostratigraphic agreement (Supplementary Fig. 3).

### Identification of the wood

To facilitate comprehensive morphological descriptions and taxonomic identifications, each of the two main sticks (XU8–9: 10,720–12,420 cal BP and XU11: 11,420–12,950 cal BP; Bayesian-modelled ages) associated with the miniature fireplaces in square R31 at Cloggs Cave was oriented in accordance with its distinct anatomical features. This orientation anatomically designates the proximal end as the segment nearest to the root stock, while the distal end is positioned at the farthest point along the stick. These observations are anchored in the discernible features, such as the proximal end displaying a larger count of annual growth rings and boasting a larger diameter in comparison to the distal end. Furthermore, the base of most small twigs emerging from the main stem of each stick exhibits a characteristic inclination away from the proximal end, as correctly expected from the stick’s orientation relative to the original root stock. A major proportion of the small branch growths on both the XU8–9 and XU11 sticks conform to this uniform orientation, all directed towards the distal end. This alignment converges with the ring count and diameter observations.

The anatomical orientation terms employed herein, notably ‘proximal’ and ‘distal’, may not align with the archaeological orientation of the sticks’ original recovery contexts. That is, ‘proximal’ does not denote the end nearest to the fire, but to its anatomical orientation as described above.

Preliminary external observations of the sticks’ surface characteristics reveal several distinctive attributes. These encompass the presence of bark, white limestone or calcium carbonate accretion, trimmed twigs and certain discernibly darkened areas. Notably, the darkened regions are of particular interest as they may arise from various causes. These may encompass post-depositional discolouration; the adherence of fats, oils or minerals to the surface; or exposure to burning or heat. Upon closer scrutiny, these darkened areas unveil tell-tale signs of charcoalification—an alteration induced by heat exposure. This charcoalification appears ephemeral in nature: on each of the two sticks, it extends to a depth of only 1–2 mm below the surface. On neither stick does charcoalification exhibit the more extensive transformations associated with prolonged heat exposure, such as vitrification or complete carbonization. The areas manifesting the signs of ephemeral burning or heat exposure extend to roughly 50 mm from the distal end of each stick. A distinct 30-mm-long section at the proximal end of the XU11 stick exhibits evidence of a more transient degree of burning or heat exposure compared with the distal end.

Each stick also bears tell-tale signs of small branch segments that have been trimmed flush with the stick surface (for example, Fig. 4c–g). These segments appear to have undergone deliberate cutting or slicing, as evidenced by their remarkably smooth and square-cut terminations. There are no clearly distinguishable cut marks from the tools used.

#### Anatomical observations on the two trimmed sticks

A comprehensive analysis of the sticks’ internal wood structures was undertaken by examining a small fragment that had separated from each of the sticks during excavation and subsequent handling and storage. To identify the taxon of each stick based on its internal anatomical features, 163 anatomical characteristics that occur across the three distinct anatomical planes—transverse, tangential and radial—were examined and described, as stipulated in the International Association of Wood Anatomists’ list of microscopic features for hardwood identification^28^. Not all of these characteristics are diagnostic in every instance. Rather, the combination of characteristics yields a reliable identification, usually to genus level and in some instances to species level. For Australian arboreal taxa, considerable overlap is often evident in many anatomical features. This complicates the process of identification and, for many taxa, means that wood identification cannot be undertaken to species level.

Each analysed wood sample was examined under reflected light microscopy to determine its internal wood structure. The wood anatomy of each of the two sticks exhibits characteristics consistent with those of Dicotyledons (flowering trees) as opposed to Gymnosperms (conifers) and Monocotyledons (grasses).

##### Transverse plane

On the transverse section, conspicuous features of each stick include large multi-seriate rays (>10 cells wide), two to four seriate rays and uni-seriate rays. The majority of rays are heterogeneous uni-seriate (Supplementary Fig. 4a), interspersed with occasional aggregate multi-seriate rays occurring approximately every 4–8 mm (Supplementary Fig. 4a,b). Additionally, there are multiple but sporadic smaller multi-seriate rays present, typically comprising three to four seriate arrangements. Prismatic crystals are discernible in both the procumbent ray cells and the axial parenchyma cells.

The vessels visible along the transverse plane are predominantly solitary, with a frequency for solitary alignment exceeding 95% (Supplementary Fig. 4a,b). They do not share vessel walls and are not organized in clusters or files. These vessels exhibit diffuse porosity, characterized by an absence of a consistent transition in vessel size from early to late wood. There is a minor tendency towards semi-diffuse porosity and in some rare instances, semi-ring porous. The size range of vessels in the semi-ring-porous wood is relatively small and displays inconsistency when present, with vessels typically falling into the small to medium-sized category (mean tangential diameter of 30–80 µm, as seen in Supplementary Fig. 4b). The parenchyma is diffuse in aggregates, often appearing in tangential files, typically spanning two to four cells in width.

##### Tangential and radial planes

The majority of perforation plates are simple, occasionally exhibiting scalariform characteristics. Rare spiral thickenings are observed in the vessels. Tracheid pits are found in alternate and opposite arrangements when bordering the vessel, forming a sheath around the vessel that mirrors vessel wall thickenings. The ground tissue primarily comprises fibre tracheids, featuring conspicuously bordered pits that are typically present on both tangential and radial walls. The vessel-ray pits are regularly shaped and have clear borders.

##### General growth observations

Each of the two sticks probably originated from an upright stem, as opposed to a branch or root wood. This conclusion is drawn from the uniformity of the wood around the central or core portion of each stick (Fig. 4b). Branches and stem wood typically develop compression and tension wood in response to mechanical and gravitational forces, thereby shifting the core and causing non-uniform growth around it. In contrast, the observed uniformity in each of the two archaeological sticks from square R31 at Cloggs Cave implies their origin from wood sections that were growing upright. This choice aligns with mechanical and functional considerations, as upright stems tend to offer better balance and uniform strength.

##### Identification

The International Association of Wood Anatomists (IAWA) identification criteria allow separation between the *Allocasuarina* and *Casuarina* genera on the basis of several factors. First, *Allocasuarina* wood has both heterocellular and homocellular ray composition, with heterocellular cells occurring in both procumbent form and square/upright form. Such heterocellular composition with square and upright cells is absent among the *Casuarina* spp.^29^. Second, the very small vessels often present in *Allocasuarina* spp. are also absent in *Casuarina* spp., which have two distinct sizes rather than the three that occur among the *Allocasuarina* spp.^29^. Third, the helical thickenings that occur in *Allocasuarina* spp. vessels are absent from the *Casuarina* spp.

The two Cloggs Cave square R31 sticks anatomically match the Casuarinaceae, specifically *Casuarina cf. cunninghamiana*. It is more difficult to separate different species of *Casuarina* from their wood anatomy, hence the *cf*. designation. For the most part, the observations undertaken best fit with *Casuarina cf. cunninghamiana* or *C.* *glauca*. *C.* *cunninghamiana* and *C.* *glauca* share numerous anatomical similarities. However, both species can be distinguished from *Casuarina equisetifolia*, primarily due to the latter’s tendency to exhibit semi-diffuse or semi-ring-porous characteristics^30^. Additionally, *C.* *equisetifolia* and *C.* *glauca* both typically feature rays that are mostly uni-seriate, two to three seriate and rarely more than four seriate. In contrast, *C.* *cunninghamiana* is characterized by numerous multi-seriate and aggregate rays (Supplementary Fig. 1). Although a number of anatomical observations of the two archaeological sticks from Cloggs Cave closely match those of *C.* *equisetifolia*, the number of elements absent (as listed in Supplementary Table 3) implies that the stick is not *C.* *equisetifolia* and is most probably *Casuarina cf. cunninghamiana* or possibly *C.* *glauca*.

#### General observations

On the basis of these observations alone, two potential scenarios emerge as the most likely functions of the two trimmed sticks from Cloggs Cave square R31.

First, each stick was used to tend or stoke a small fire of low-heat intensity. The presence of partially burnt or charcoalified areas on each stick indicates very brief exposure to heat. The extent of charcoalification is limited, suggesting that the stick either fell out of the fire prematurely or was intentionally removed from the flames before complete combustion or transformation into charcoal. This pattern of burning is consistent with a tool used to manipulate a fire, which necessitates a certain level of preservation to fulfil its intended function. In such a scenario, the trimming of smaller twigs from the stick’s surface would serve the purpose of rendering it smoother to enhance its utility in tending or poking the fire. Such an act of twig removal would suggest a level of care and intent regarding the stick’s preparation for its fire-related task.

An alternative interpretation emerges. Rather than being intended for full consumption by the fire, each stick was deliberately shaped or modified for a specific fire-related role. The trimming of small twigs from the parent stick, as noted above, served the dual purpose of smoothening the stick surface and preparing it for its intended function. This scenario implies that the stick was crafted or chosen with a particular purpose in mind, distinct from mere fuel or temporary stoking for the fire. It is worthwhile noting that no other stick of similar dimensions occurs in either of the two archaeological fires reported here, and in neither fire is charcoal abundant.

Apart from the removal of smaller twigs, no other manipulation of the sticks’ shape or surface is discernible from the wood itself. This implies a certain level of simplicity in the sticks’ modification, which can be attributed to the pragmatic requirements of their intended function. While these observations offer insights into the sticks’ potential use(s), contextual archaeological and residue analyses (see below) shed additional light on how they were used.

### Residue analysis

Aseptic practices were employed during all stages of use-wear analysis. This included pre-cleaning the Dino-Lite microscope stand base and Leitz Dialux slide holder with ethanol using Kimtech 34120A Science brand Kimwipes. The Dino-Lite stand base was then covered with the plastic bag used to house each wooden object, one at a time. At all times during analyses, hands were washed and gloved with Halyard Sterling Nitrile-Xtra powder-free Exam gloves with extended cuffs, and benches were cleaned with ethanol and the laboratory space was kept closed. The wooden artefacts were examined under low-power magnification utilizing a Dino-Lite AM 4815ZT microscope with polarizing capability. A combination of plane, cross-polarized and oblique lighting was used to examine all surfaces and faces of each of the two sticks at magnifications of ×25 to ×85. The Dino-Lite was used to guide focused extractions from identified areas of interest across the wood surfaces. Ultra-purified water (not a solvent) was used as an extraction medium. No extraction solvent was used, as solvents frequently dissolve residues and we wanted to preserve them. The ultra-purified water was expelled and suctioned through a small, sealed pipette tip to create hydrostatic pressure. This pressure or force acts like a Venturi effect and removes all residues into the aqueous solution, extracting the sample in the process^31^. Each ultra-purified water extraction sample was placed on a pre-cleaned microscope slide and then underwent adapted Picrosirius Red (PSR) staining practices^32^. High-powered microscopy (×200–400) with a Leitz Dialux 22 microscope with polarizing capability was used to examine the stained slides. Observed residues were photographed with a Tucsen ISH 500 camera in plane, part-polarized and cross-polarized light at magnifications of ×250 and ×400. For this study, four samples were extracted from each of the two archaeological sticks.

#### Results

The key finding was the presence of lipids (fatty residues) associated with four of the extractions. This included adipose/lipid tissue fragments across extraction 1 from the XU11 stick; a lipid-like mass across extraction 3 from the XU11 stick; lipid smears across extraction 3 from the XU8–9 stick; and lipid smears across extraction 4 from the XU8–9 stick. Of note, the highest density of lipid material was the extensive and numerous lipid smears associated with extraction 3 from the XU8–9 stick. The sample was extracted from a discrete area displaying a patch of light colour change compared with the body of wood that was outlined by charring or carbonized material (Fig. 3b). This change in colour on the stick had been clearly observed when the stick was still in situ during its excavation. While lipids are able to be extracted using the Venturi effect outlined above, they will not absorb the water-based PSR stain. Generally, lipids can be seen as clear transparent areas with droplet features similar to bubbles and a defined irregular margin, as is also the case when fat is smeared on a glass surface. Lipids can be differentiated from plant cellulose in cross-polarized light; rather than fluoresce, the lipid hazes and in some cases can disappear completely. Lipids can be associated with plant or animal matter or waxes. We note that while *Casuarina* spp. trees contain a volatile oil within their leaves, lipid material is not associated with the woody structures of the plant. As such, the lipid material noted in the Cloggs Cave archaeological sticks sampled here could not have originated from the wood itself and it is too abundant on the wood surface to have been incidentally contaminated by brushing leaves. Furthermore, the discrete locations where the lipid residues were found indicates that the lipid or fatty material was added/applied to the surface of the wood.

### Lipid analysis

The lipid analyses followed directly the residue analysis described above. The residue was removed from the glass slide containing the extraction using acetonitrile (LC–MS-grade, Sigma-Aldrich) by applying 200 µl to the surface and removal using a pipette. The samples were extracted from the slides rather than newly sampled from the sticks themselves because the sticks had undergone conservation treatment after initial sampling for the slides. The removed solution was placed into a 2 ml sterile glass autosampler phial and showed a slight yellow colour. An aliquot of 50 µl was taken from this solution and prepared for GC–MS using a TSQ 9000 (Thermo Scientific) system, while the remaining 150 µl was retained for LC–MS using an Exploris 120 Orbitrap (Thermo Scientific) mass spectrometer.

#### Analysis using LC–MS

The samples were directly applied to LC–MS on the Exploris 120 Orbitrap. A 2.5 µl injection volume was used with a static spray voltage and gas mode. The column temperature was 50 °C. The runs were in positive polarity with the Orbitrap resolution of 60,000 and a scan range from 85 to 950 *m*/*z*. A 12-min run time was used. Two mobile phase solutions were used, a 1% formic acid in water (solution A) and 100% acetonitrile (solution B). The solution run conditions were set to 0–7.5 min using a 5% solution B, 7.5–10 min using 95% solution B and 10–12 min back with 5% solution B. The data generated were analysed using Xcaliber software and compound identification was performed using the Compound Discoverer v3.2 software with mzCloud, ChemSpider, mzVault and MassList databases.

#### Analysis using GC–MS

The extract solution was derivatized for GC–MS analysis. A 50 µl volume of extract solution was placed into a new 2 ml sterile glass autosampler phial. A volume of 10 µl of bis(trimethylsilyl)trifluoroacetamide with 1% trimethylsilyl was added to the extract solution, purged with nitrogen, sealed and heated at 120 °C for 25 min. This solution was made up to 100 µl using LC–MS-grade acetonitrile. The samples were run on the TSQ 9000 GC–MS system. An injection volume of 1 µl was used and the run time was 23.49 min. The data were analysed using the Chromeleon software.

#### Results

The LC–MS analysis of lipids and fatty acids generated a large number of fatty acids and glycerides from the residue taken from the wooden artefact. Most of these compounds were supported by the GC–MS results. Three intact saturated fatty acids (capric acid, pentadecanoic acid and palmitic acid) were identified from the residue (Supplementary Table 4 and Supplementary Fig. 5). Seven monounsaturated fatty acids (nonenoic acid, 2-decenoic acid, undecylenic acid, myristoleic acid, palmitoleic acid, oleic acid and erucic acid) were also identified (Supplementary Table 4 and Supplementary Fig. 5). Six polyunsaturated fatty acids (linoelaidic acid, α-linolenic acid, linolenelaidic acid, parinaric acid, arachidonic acid-d11 and adrenic acid) were also identified in this analysis (Supplementary Table 4 and Supplementary Fig. 5). In addition to the fatty acids, 13 glycerides and glycerol were also found in this residue sample. Glycerides are lipids that are made up of between one to three fatty acids and glycerol. There were three triglyceride with combinations of lauric acid, caprylic acid, capric acid, stearidonic acid and behenic acid. Diglycerides with caprylic, palmitoleic acid and oleic acid are present, as are four monoglycerides of linoleic acid, tridecylic acid, stearic acid and palmitic acid.

In addition to these fatty acids and lipids, many compounds that show evidence of modification and degradation were also identified. These include dicarboxylic acids, fatty amides, fatty acid methyl esters and oxidation products of fatty acids. The amount of these degraded and modified fatty acids indicates the abundance of the original fatty acids that may have been present in the residue. The most common dicarboxylic acid breakdown products from the degradation of unsaturated fatty acids (sebacic acid, adipic acid and pelargonic acid) were identified in this residue. These modified fatty acids include the oxidation of palmitic acid and amination of capric acid, lauric acid, myristic acid, palmitic acid, stearic acid, oleic acid, arachidic acid, behenic acid and tricosylic acid.

Some of the fatty acids identified in this analysis are found in animal and human fats, especially palmitic acid, stearic acid, oleic acid, linolenic acid, myristic acid, palmitoleic acid, margaric acid and linoleic acid^33–36^. Although individually these can also be found in plant and microbial sources, the combinations and relative abundance found in this residue excludes these other sources. All of these compounds are found in animal and human fat.

### Reporting summary

Further information on research design is available in the Nature Portfolio Reporting Summary linked to this article.

### Supplementary information

Supplementary Information Supplementary Figs. 1–5 and Tables 1–4.

Reporting Summary

## Data Availability

The authors confirm that all data generated or analysed during this study are included in this published article. The InsideWood Database 2004 (available at http://insidewood.lib.ncsu.edu/search) was used in this study. Additional primary data on excavation depths described in this study are available in refs. ^13,19^. The primary data on the two stone artefacts associated with the XU8–9 fireplace are available in ref. ^15^. The primary data on the radiocarbon dates are available in Supplementary Tables 1 and 2. The archaeological materials described in this paper are currently stored in the Monash Indigenous Studies Centre archaeology laboratories and will be returned to the GunaiKurnai Land and Waters Aboriginal Corporation upon completion of the study.
