# Supplementary material for: Archaeological evidence of an ethnographically documented Australian Aboriginal ritual dated to the last ice age
Source: Nat Hum Behav. 2024 Jul 1;8(8):1481–92. doi: 10.1038/s41562-024-01912-w (PMC11343701; doi:10.1038/s41562-024-01912-w)
Supplement: Supplementary file 1 — Supplementary Figs. 1–5 and Tables 1–4. [file 41562_2024_1912_MOESM1_ESM.pdf]

nature human behaviour

Supplementary information

https://doi.org/10.1038/s41562-024-01912-w

Archaeological evidence of an ethnographically documented Australian Aboriginal ritual dated to the last ice age

In the format provided by the authors and unedited

Supplementary Information

Supplementary Figures

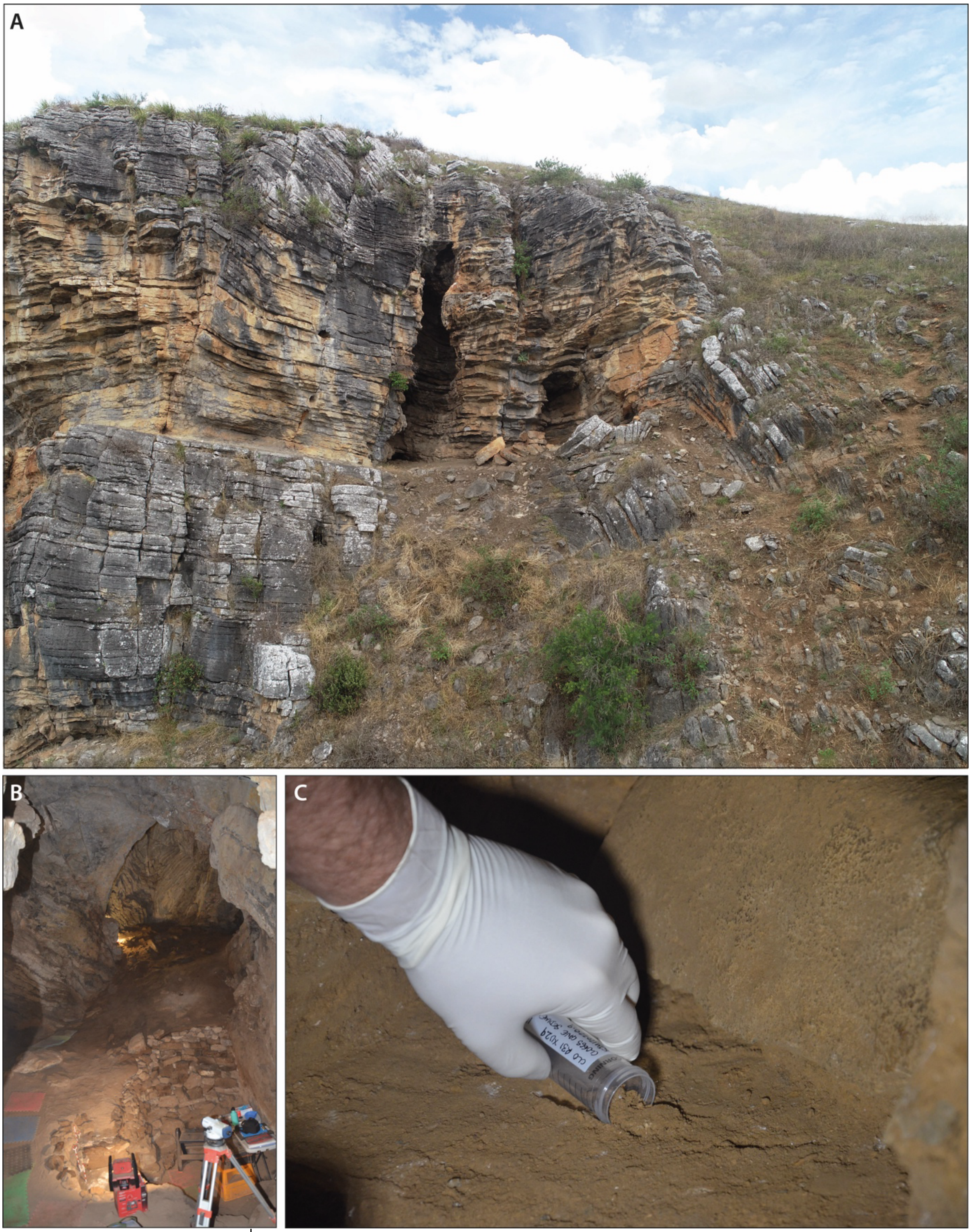

Supplementary Fig. 1 │ Cloggs Cave. A, Entrance to the cave. B, inside the cave, excavation of square R31 (left extension of the main pit) in progress. C, Collecting sedaDNA sediment sample from square R31 prior to commencing excavation of XU29.

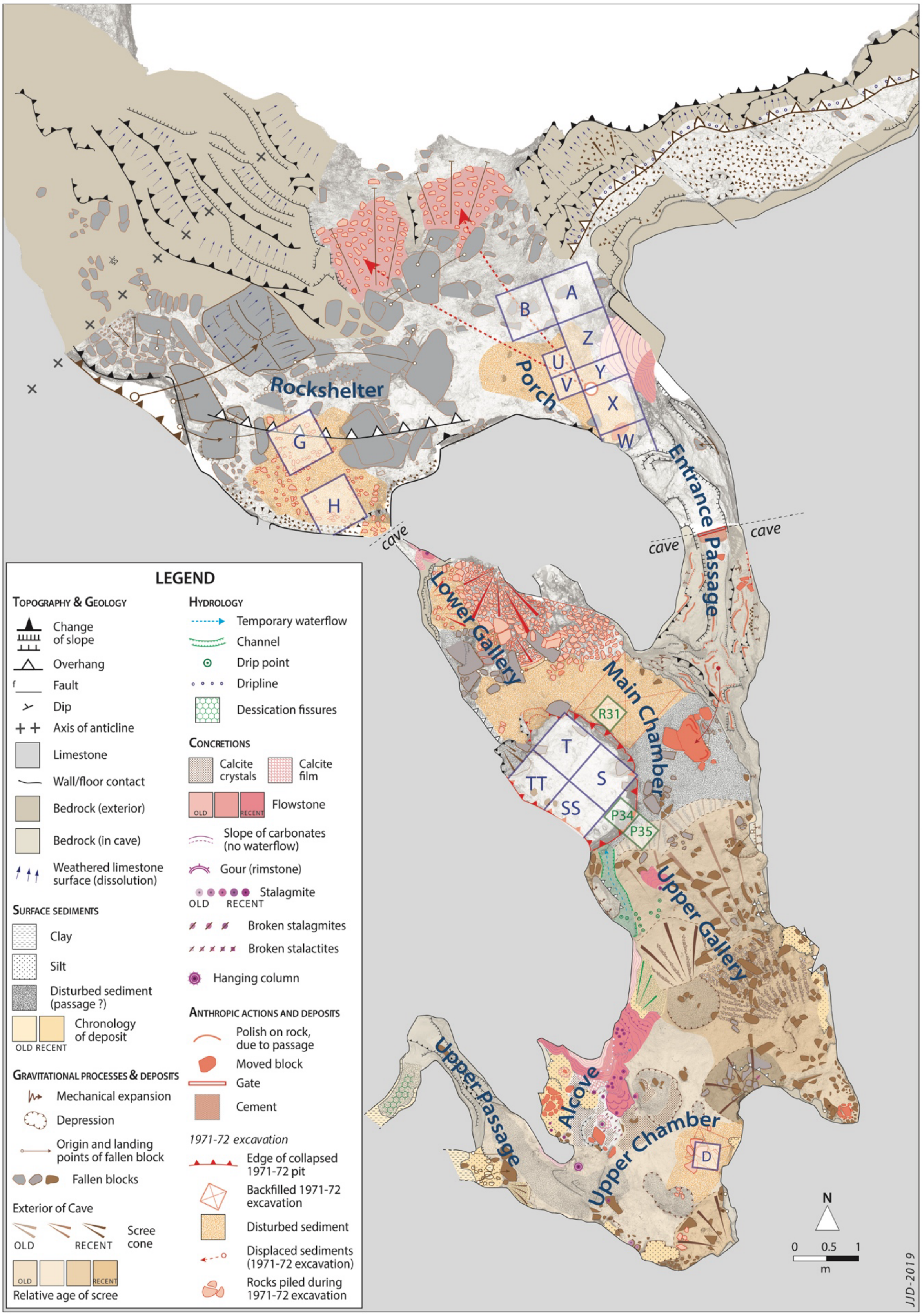

Supplementary Fig. 2 | Geomorphological map of Cloggs Cave, showing the location of the 1971–1972 and 2019–2020 excavation squares.

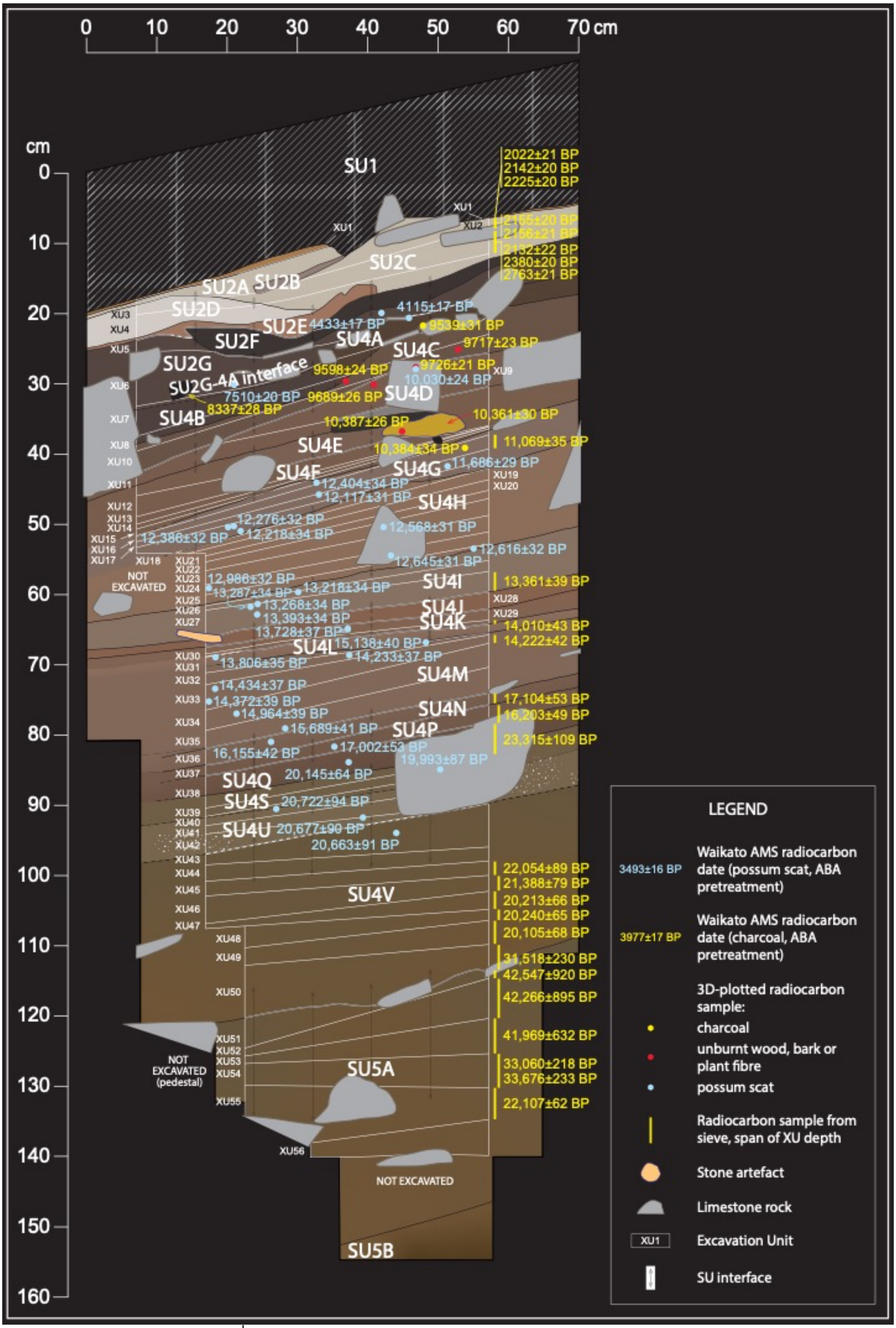

Supplementary Fig. 3 | Section drawing of Cloggs Cave square R31, with all the AMS radiocarbon dates back-plotted.

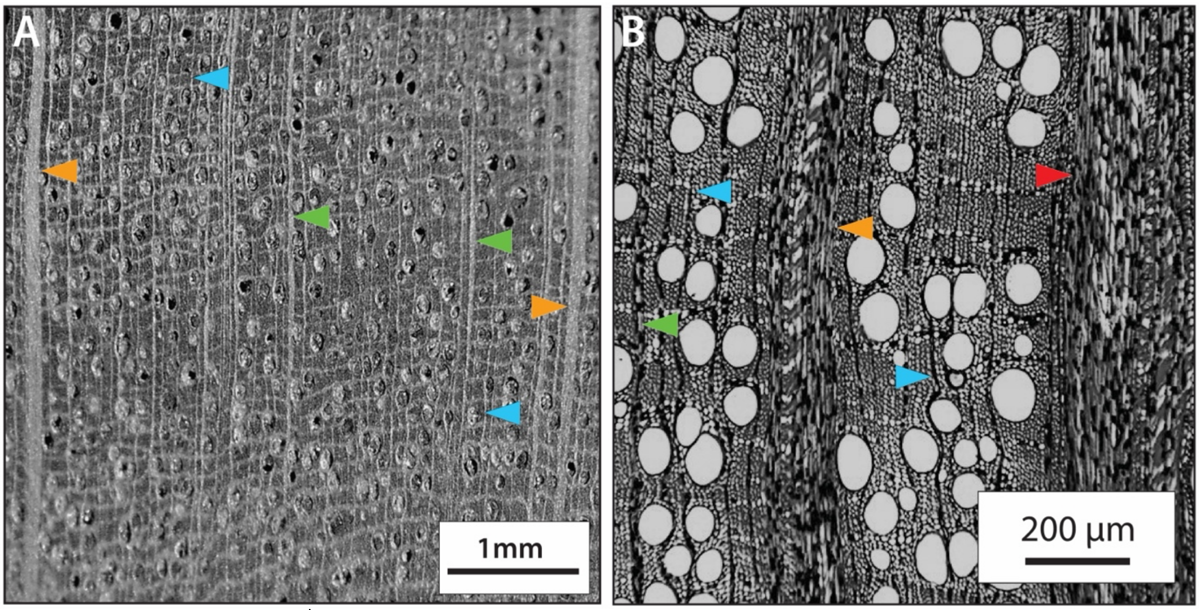

Supplementary Fig. 4 │ Transverse views of the vessel structure of the two trimmed sticks from Cloggs Cave square R31. A, Transverse section of the XU11 stick, showing large multiseriate rays (orange arrows), uniseriate rays (blue arrows), and 2–4-seriate rays (green arrows), along with solitary vessels of two sizes (small and medium, dominated by medium). Photography undertaken at ×10 magnification. B, Transverse thin-section of the XU8–9 stick showing solitary vessels and large multiseriate rays (orange arrow) alongside 2–4-seriate rays (green arrow) and uniseriate rays (blue arrows). Large aggregate ray depicted by red arrow. Note parenchyma is present in narrow bands up to 3 cells wide. Photography undertaken at ×100 magnification.

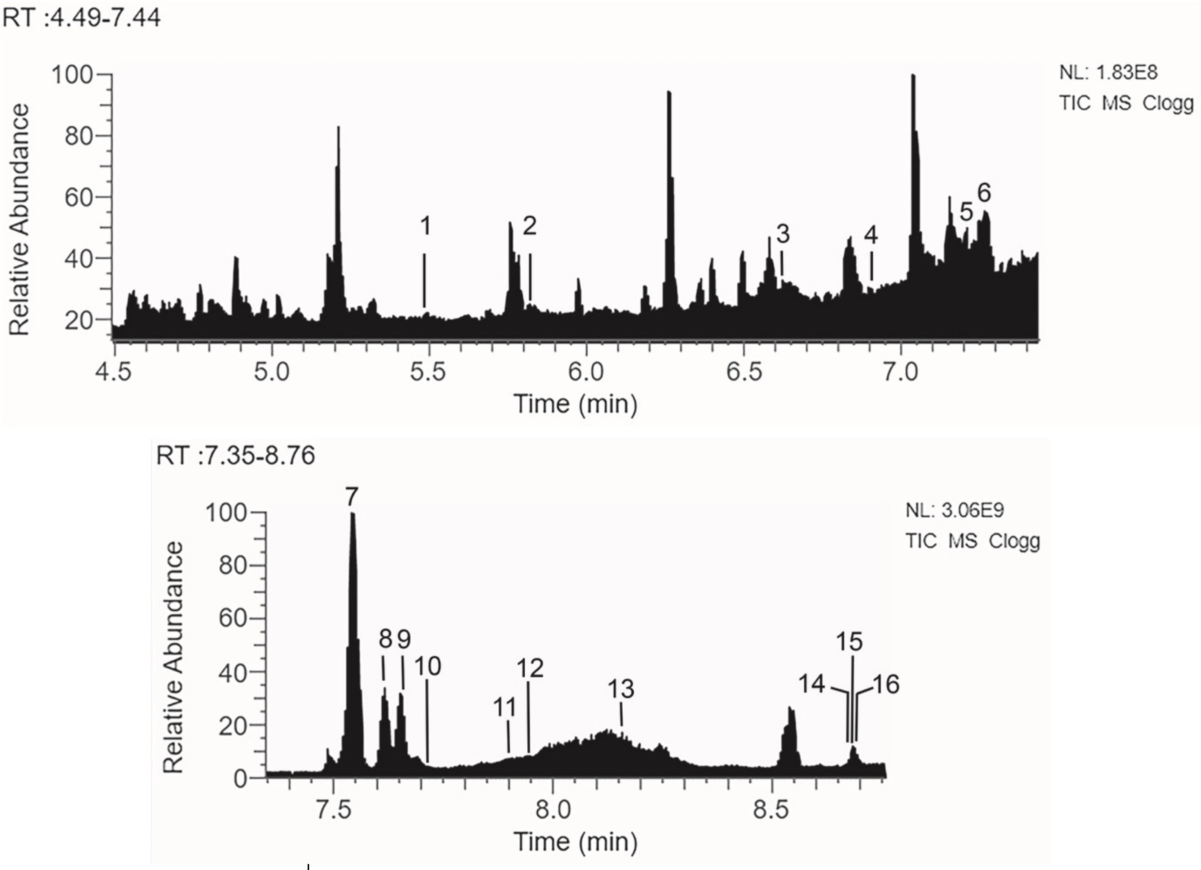

Supplementary Fig. 5 │ Chromatogram of the chemical analysis of the lipid residue. Many compounds were recovered from the residue. The fatty acids identified here are: 1) pentadecanoic acid; 2) capric acid; 3) 2-decenoic acid; 4) nonenoic acid; 5) palmitoleic acid; 6) α-linolenic acid; 7) oleic acid; 8) linolenelaidic acid; 9) myristoleic acid; 10) parinaric acid; 11) palmitic acid; 12) adrenic acid; 13) arachidonic acid; 14) erucic acid; 15) linoelaidic acid; 16) undecylenic acid.

Supplementary Tables

Supplementary Table 1 │ AMS radiocarbon ages on individual fragments from square R31, Cloggs Cave. * Collected from the wall of the cleaned exposed 1971–1972 pit prior to commencement of the square R31 excavation (i.e., this sample does not have an XU attribution) (after David et al. 2021: Table 2). † Dated sample comes from the sieves, from a level that includes rocky SU4V–SU5A sediments mixed from roof-fall.

SU	XU	Material Dated	Wk- Laboratory Code	$\delta^{13}C$ (‰)	^{14}C Age (BP)	%C
2A-2C	1	charcoal	51363	-27.0 ± 0.7	2022 ± 21	55
2A-2C	1	charcoal	51364	n/a	2142 ± 20	55
2A-2C	1	charcoal	51365	-24.1 ± 0.7	2225 ± 20	67
2A-2C	2	charcoal	51366	-23.4 ± 0.7	2155 ± 20	51
2A-2C	2	charcoal	51367	-26.5 ± 0.7	2156 ± 21	56
2C-2D	3	charcoal	51368	-24.9 ± 0.7	2132 ± 22	50
2C-2D	3	charcoal	51370	-24.5 ± 0.7	2380 ± 20	63
2C-2D	3	charcoal	51369	-25.8 ± 0.7	2763 ± 21	54
2F-2G interface	4	possum scat	50961	n/a	4433 ± 17	n/a
2F-2G interface	4	possum scat	50962	n/a	4115 ± 17	n/a
2G-4A interface	6	possum scat	50963	n/a	7510 ± 20	n/a
2G-4A interface	*	charcoal	50276	-24.1 ± 0.5	8337 ± 28	46
4A-4C interface	7	charcoal	50964	-24.1 ± 0.4	9539 ± 31	65
4C	8	bark	50965	-22.4 ± 0.4	9717 ± 23	n/a
4C	8	softwood plant fibre	50966	-24.7 ± 0.4	9598 ± 24	n/a
4C	8	twig	50967	-20.9 ± 0.9	9689 ± 26	n/a
4C	9	softwood artefact	50968	-21.6 ± 0.9	9726 ± 21	n/a
4C-4D interface	9	possum scat	50969	-19.4 ± 0.9	10,030 ± 24	n/a
4E	11	wooden artefact	50278	-24.3 ± 0.1	10,361 ± 30	n/a
4E	11	bark	50970	n/a	10,387 ± 26	n/a
4E-4F interface	12	charcoal	50971	-23.0 ± 0.9	10,384 ± 34	66
4F-4G interface	16	charcoal	51126	-23.5 ± 0.2	11,069 ± 35	75
4F-4G interface	15	possum scat	51036	-27.6 ± 0.4	11,686 ± 29	n/a
4G	16	possum scat	51037	-23.6 ± 0.4	12,404 ± 34	n/a
4G	17	possum scat	51038	-23.4 ± 0.4	12,117 ± 31	n/a
4G	18	possum scat	51039	-24.0 ± 0.4	12,386 ± 32	n/a
4G	19	possum scat	51040	-17.9 ± 0.4	12,276 ± 32	n/a
4G	20	possum scat	51041	n/a	12,218 ± 34	n/a
4H	21	possum scat	51042	-25.9 ± 0.5	12,568 ± 31	n/a
4H	22	possum scat	51043	-21.8 ± 0.5	12,616 ± 32	n/a
4H	23	possum scat	51044	-19.3 ± 0.5	12,645 ± 31	n/a
4H	24	possum scat	51045	-24.7 ± 0.5	12,986 ± 32	n/a
4I	25	possum scat	51046	-27.1 ± 0.5	13,218 ± 34	n/a
4I	26	possum scat	51047	-12.8 ± 0.5	13,268 ± 34	n/a
4I	27	possum scat	51048	-16.4 ± 0.5	13,287 ± 34	n/a
4I	27	charcoal	51127	n/a	13,361 ± 39	74
4I	28	possum scat	51049	-23.3 ± 0.5	13,393 ± 34	n/a
4K	29	possum scat	51050	-21.4 ± 0.5	13,728 ± 37	n/a
4K-4L interface	30	possum scat	51051	-18.9 ± 0.5	13,806 ± 35	n/a
4K-4L interface	30	charcoal	51128	-24.9 ± 0.2	14,010 ± 43	73
4L-4M interface	31	possum scat	51052	-24.4 ± 0.5	15,138 ± 40	n/a
4L-4M interface	32	possum scat	51053	n/a	14,233 ± 37	n/a
4M	33	charcoal	51129	-23.8 ± 0.2	14,222 ± 42	75
4M	33	possum scat	51054	-24.8 ± 0.5	14,434 ± 37	n/a
4M	34	possum scat	51055	-23.0 ± 0.5	14,372 ± 39	n/a

4M	35	possum scat	51056	n/a	14,964 ± 39	n/a
4M-4N interface	36	possum scat	51057	-28.5 ± 0.5	15,689 ± 41	n/a
4N	37	possum scat	51059	n/a	16,155 ± 42	n/a
4N-4P interface	37	charcoal	51131	-23.0 ± 0.2	16,203 ± 49	73
4M-4N-4O-4P interface	36	charcoal	51130	-22.1 ± 0.6	17,104 ± 53	72
4P	37	possum scat	51058	-23.2 ± 0.5	17,002 ± 53	n/a
4Q	38	possum scat	51060	n/a	20,145 ± 64	n/a
4Q	38	charcoal	51132	-21.1 ± 0.6	23,315 ± 109	n/a
4Q-4R-4S interface	39	possum scat	51133	n/a	19,993 ± 87	n/a
4S-4U interface	40	possum scat	51134	-29.2 ± 0.4	20,722 ± 94	n/a
4U	41	possum scat	51135	n/a	20,677 ± 90	n/a
4U	42	possum scat	51136	-19.9 ± 0.4	20,663 ± 91	n/a
4V	45	charcoal	51138	n/a	22,054 ± 89	72
4V	46	charcoal	51139	n/a	21,388 ± 79	68
4V	47	charcoal	51140	n/a	20,213 ± 66	71
4V	48	charcoal	51141	n/a	20,240 ± 65	75
4V	49	charcoal	51142	n/a	20,105 ± 68	68
4V-5A interface	50	charcoal	51143	n/a	31,518 ± 230	72
5A	51	charcoal	51144	n/a	42,547 ± 920	68
5A	52	charcoal	51145	n/a	42,266 ± 895	71
4V-5A interface	53	charcoal	51190	-24.3 ± 0.6	41,969 ± 632[†]	72
4V-5A interface	54	charcoal	51191	-21.9 ± 0.6	33,060 ± 218[†]	68
4V-5A interface	54	wood	51192	-23.5 ± 0.6	33,676 ± 233[†]	n/a
4V-5A interface	55	charcoal	51193	-27.1 ± 0.6	22,107 ± 62[†]	71

Supplementary Table 2 | Results of the Bayesian sequence model. Rows highlighted in dark grey represent boundary ages (after David et al. 2021: Supplementary Table 3). For details of the Bayesian modelling, see David et al. (2021).

Name	Unmodelled (BP)				Modelled (BP)				
	68.2% Prob.		95.4% Prob.		68.2% Prob.		95.4% Prob.		Convergence
**Cloggs Cave End**					1990	1800	2020	1460	77
	**SU2A-2D**								
**Wk-51363 (2022,21)**	2000	1890	2000	1880	2000	1910	2010	1880	93.8
**Wk-51364 (2142,20)**	2100	2010	2130	2000	2100	2010	2130	2000	99.7
**Wk-51365 (2225,20)**	2310	2120	2320	2090	2310	2110	2320	2090	81.6
**Wk-51366 (2155,20)**	2120	2010	2290	2010	2120	2020	2290	2010	99.7
**Wk-51367 (2156,21)**	2120	2010	2290	2010	2120	2050	2290	2010	99.7
**Wk-51368 (2132,22)**	2090	2010	2120	2000	2090	2010	2120	2000	99.8
**Wk-51369 (2763,21)**	2850	2770	2880	2750	2880	2040	2930	1890	0.1
**Wk-51370 (2380,20)**	2410	2180	2470	2150	2370	2150	2490	2150	50.8
**Boundary SU2D end**					3040	2180	3360	2130	0.5
**Boundary SU2F start**					4770	3820	4800	2950	98
	**SU2F-2G interface**								
**Wk-50962 (4115,17)**	4790	4450	4810	4440	4800	4450	4820	4430	99.8
**Wk-50961 (4433,17)**	5040	4870	5220	4860	5030	4870	5260	4850	99.8
**Boundary SU2F-2G Interface start**					5780	4890	7290	4860	99.5
**Boundary SU2G-4A Interface end**					8330	6970	9300	5310	92.7
	**SU2G-4A interface**								
**Wk-50276 (8337,28)**	9420	9150	9440	9130	9420	9140	9460	9120	99.5
**Wk-50963 (7510,20)**	8350	8200	8370	8190	8360	8200	9520	8170	97.8
**Boundary SU4A-4C end**					10760	9380	10990	9290	99.3
**SU4A-4C Wk-50964 (9539,31)**	11070	10690	11070	10590	11070	10680	11080	10580	99.7
**Boundary SU4C-4D Interface end**					11470	10860	11580	10720	99.8
**SU4C-4D interface Wk-50969 (10030,24)**	11620	11320	11630	11270	11630	11370	11630	11270	99.9
**Boundary SU4E end**					12140	11720	12420	11420	99.6
	**SU4E**								
**Wk-50970 (10387,26)**	12440	12020	12470	11990	12430	12010	12450	11970	99.8
**Wk-50278 (10361,30)**	12430	11960	12440	11930	12430	11990	12440	11940	99.8
**SU4E/4F Interface Wk-50971 (10384,34)**	12440	12010	12470	11960	12430	12010	12460	11970	99.9
**Boundary SU4F-4G Interface end**					12600	12080	12950	12040	99.2
	**SU4F-4G Interface**								
**Wk-51036 (11686,29)**	13580	13460	13600	13440	13580	13460	13600	13440	99.8
**Wk-51126 (11069,35)**	13070	12920	13090	12850	13070	12920	13090	12840	99.7
**Boundary SU4G end**					14040	13750	14060	13550	99.9

	SU4G								
Wk-51041 (12218,34)	14130	14040	14300	13880	14130	14040	14310	13880	99.9
Wk-51040 (12276,32)	14190	14070	14320	14050	14190	14070	14310	14060	99.9
Wk-51039 (12386,32)	14790	14200	14830	14140	14400	14170	14790	14110	99.9
Wk-51038 (12117,31)	14060	13860	14070	13800	14070	13870	14080	13810	99.8
Wk-51037 (12404,34)	14800	14240	14840	14170	14440	14200	14800	14130	99.9
Boundary SU4H end					14850	14300	14930	14240	99.9
	SU4H								
Wk-51045 (12986,32)	15590	15390	15640	15300	15570	15370	15640	15290	99.8
Wk-51044 (12645,31)	15110	14960	15180	14870	15110	14960	15190	14860	99.8
Wk-51043 (12616,32)	15090	14910	15150	14610	15090	14920	15160	14630	99.8
Wk-51042 (12568,31)	15000	14610	15080	14520	15020	14650	15100	14540	99.8
Boundary SU4I end					15890	15630	15960	15460	99.8
	SU4I								
Wk-51049 (13393,34)	16160	15990	16240	15900	16120	15950	16200	15860	99.9
Wk-51127 (13361,39)	16120	15930	16200	15840	16080	15910	16170	15830	99.9
Wk-51048 (13287,34)	15990	15830	16070	15750	16000	15850	16060	15770	99.9
Wk-51047 (13268,34)	15970	15800	16040	15730	15980	15830	16040	15750	99.9
Wk-51046 (13218,34)	15900	15730	15980	15670	15950	15780	16000	15710	99.9
Boundary SU4K-4L Interface end					16290	15970	16550	15910	99.7
	SU4K-4L Interface								
Wk-51128 (14010,43)	17070	16940	17230	16760	17060	16880	17100	16660	99.7
Wk-51051 (13806,35)	16800	16580	16920	16510	16800	16580	16920	16500	99.9
Wk-51050 (13728,37)	16650	16450	16750	16360	16650	16450	16770	16360	99.9
Boundary SU4L-4M Interface end					17160	16940	17260	16770	99.9
	SU4M/N/O/P								
end					17260	17080	17350	16990	99.5
	SU4L-4M Interface								
Wk-51053 (14233,37)	17340	17130	17390	17090	17290	17130	17350	17090	99.3
Wk-51052 (15138,40)	18610	18270	18650	18240	17300	17130	17370	17060	99.1
Boundary SU4 end					17350	17190	17390	17110	98.6
	SU4M								
Wk-51056 (14964,39)	18260	18200	18300	18150	18270	18190	18580	17260	95.5
Wk-51055 (14372,39)	17500	17330	17770	17290	17490	17330	17760	17300	99.2
Wk-51054 (14434,37)	17710	17400	17810	17360	17700	17380	17810	17350	95.2
Wk-51129 (14222,42)	17320	17120	17390	17080	17400	17270	17500	17120	99.1
Boundary SU4M/N Interface end					18860	18220	18970	17360	84.5
SU4M/N interface Wk-51057 (15689,41)	18950	18850	19030	18820	18950	18850	19040	18820	99.9
Boundary SU4N end					19490	19110	19510	18910	99.7
SU4N Wk-51059 (16155,42)	19540	19420	19580	19300	19520	19380	19550	19250	99.9
Boundary SU4N/P Interface end					19560	19420	19670	19260	99.9

**SU4N/P interface Wk-51131 (16203,49)**	19570	19460	19630	19340	19590	19480	19820	19410	99.9
**Boundary SU4P end**					20480	19490	20500	19480	99.2
**SU4P Wk-51058 (17002,53)**	20560	20420	20700	20330	20550	20410	20740	20280	99.7
**Start**					20900	20430	21850	20290	99.4
**SU4/M/N/O/P Wk-51130 (17104,53)**	20720	20510	20810	20470	20720	20510	20820	20460	99
**Boundary SU4P start**					22270	20690	23450	20590	99.6
**Boundary SU4Q end**					24110	23450	24210	22370	99
	**SU4Q/R/S**								
**Wk-51060 (20145,64)**	24230	24000	24290	23880	24220	24000	24300	23880	99.8
**Wk-51132 (23315,109)**	27640	27380	27730	27300	24550	23870	24880	23360	98.3
**Wk-51133 (19993,87)**	24070	23840	24200	23800	24130	23880	24230	23810	99.8
**Boundary SU4U end**					24930	24230	24990	24070	98.6
	**SU4U**								
**Wk-51134 (20722,94)**	25110	24790	25200	24630	25050	24770	25160	24630	99
**Wk-51135 (20677,90)**	25030	24720	25160	24590	25020	24750	25130	24610	99.2
**Wk-51136 (20663,91)**	25010	24700	25150	24560	25010	24740	25130	24590	99.3
**Boundary SU4V end/Rock Fall**					25330	24850	25740	24760	96.5
	**SU4V**								
**Wk-51138 (22054,89)**	26350	26070	26440	25970	26240	25950	26440	25290	88.1
**Wk-51139 (21388,79)**	25820	25660	25920	25360	25830	25660	25930	25340	99.7
**Wk-51140 (20213,66)**	24320	24070	24500	23950	26020	25200	26470	24860	92.9
**Wk-51141 (20240,65)**	24380	24110	24550	24010	26020	25190	26500	24860	95.1
**Wk-51142 (20105,68)**	24180	23950	24260	23870	26030	25200	26430	24890	91.2
**Boundary SU4V start**					26530	25980	27390	25600	67.2
**Boundary SU4V-5A interface end**					27780	25820	35810	25640	0.8
	**SU4V-5A Interface**								
**Wk-51193 (22107,62)**	26400	26090	26460	25990	29330	26030	38560	25970	0.1
**Wk-51192 (33676,233)**	39170	38220	39270	37660	39180	38160	39350	37570	97.6
**Wk-51191 (33060,218)**	38000	37020	38630	36880	38060	37020	38870	36830	97.4
**Wk-51190 (41969,632)**	45240	44290	45910	43450	44920	43290	45400	37720	92.6
**Wk-51143 (31518,230)**	36110	35600	36260	35360	36130	35590	36390	35260	97.8
**Boundary SU4V-5A Interface start**					45390	43740	45920	39310	96.3
**Boundary SU5A end**					45670	44440	46580	43230	98.2
	**SU5A**								
**OSL-1 (-44911,4150)**	51140	42580	55170	38560	46250	44590	47620	43840	99.4
**Wk-51145 (42266,895)**	45740	44340	46720	43350	45890	44790	46710	44300	99
**Wk-51144 (42547,920)**	45910	44490	47350	43840	45900	44800	46760	44350	99.1
**Boundary SU5A/B**					46550	44950	48470	44500	99.1
**OSL-2 (-49811,5510)**	57440	46080	62790	40740	48290	45240	51700	44580	99.8
**Start Cloggs Cave**					50040	45470	54160	44980	71.8

Supplementary Table 3 | List of diagnostic anatomical characteristics for *C. cunninghamiana*, *C. glauca*, and *C. equisetifolia* (after IAWA Committee (1989); InsideWood Database 2004–; Wheeler 2011; Wheeler et al. 2020, 2022; see also Moseley 1948). The grey rows indicate that the characteristics were observed in the Cloggs Cave trimmed sticks from XU8–9 and XU11. Note that growth and environmental conditions can obscure some characteristics, such as vessel porosity. The archaeological sticks from Cloggs Cave closely align with *C. cunninghamiana* and, to a lesser degree, *C. glauca*. * = only observed on the XU8–9 stick. † = only observed on the XU11 stick.

C. cunninghamiana	*C. glauca*	*C. equisetifolia*
**Growth ring boundaries distinct†**	Growth ring boundaries distinct	
**Growth ring boundaries indistinct or absent**	Growth ring boundaries indistinct or absent	Growth ring boundaries indistinct or absent
		Wood semi-ring-porous*
**Wood diffuse-porous**	Wood diffuse-porous	Wood diffuse-porous
	Vessels in diagonal and/or radial pattern	Vessels in diagonal and/or radial pattern
**Vessels exclusively solitary (90% or more)**	Vessels exclusively solitary (90% or more)	Vessels exclusively solitary (90% or more)
	Solitary vessel outline angular	
**Simple perforation plates**	Simple perforation plates	Simple perforation plates
**Scalariform perforation plates**	Scalariform perforation plates	Scalariform perforation plates
**Scalariform perforation plates with ≤10 bars**	Scalariform perforation plates with ≤10 bars	Scalariform perforation plates with ≤10 bars
**Scalariform perforation plates with 10–20 bars**		
**Inter-vessel pits alternate**	Inter-vessel pits alternate	Inter-vessel pits alternate
**Vessel-ray pits with distinct borders; similar to inter-vessel pits in size and shape throughout the ray cell**	Vessel-ray pits with distinct borders; similar to inter-vessel pits in size and shape throughout the ray cell	Vessel-ray pits with distinct borders; similar to inter-vessel pits in size and shape throughout the ray cell
**Vessels of two distinct diameter classes, wood not ring-porous**	Vessels of two distinct diameter classes, wood not ring-porous	Vessels of two distinct diameter classes, wood not ring-porous
**5–20 vessels per square millimetre**	20–40 vessels per square millimetre*	5–20 vessels per square millimetre
**20–40 vessels per square millimetre**		
		Gums and other deposits in heartwood vessels
**Vascular/vasicentric tracheids present**	Vascular/vasicentric tracheids present	Vascular/vasicentric tracheids present
**Fibres with distinctly bordered pits**	Fibres with distinctly bordered pits	Fibres with distinctly bordered pits
**Fibre pits common in both radial and tangential walls**	Fibre pits common in both radial and tangential walls	Fibre pits common in both radial and tangential walls
**Non-septate fibres present**	Non-septate fibres present	Non-septate fibres present
**Fibres very thick-walled**	Fibres thin- to thick-walled	Fibres very thick-walled
		Axial parenchyma diffuse
		Axial parenchyma diffuse-in-aggregates
**Axial parenchyma in narrow bands or lines up to three cells wide**	Axial parenchyma in narrow bands or lines up to three cells wide	Axial parenchyma in narrow bands or lines up to three cells wide
		Axial parenchyma reticulate

		Four (3–4) cells per parenchyma strand
**Eight (5–8) cells per parenchyma strand**	Eight (5–8) cells per parenchyma strand	Eight (5–8) cells per parenchyma strand
		Over eight cells per parenchyma strand
**Ray width 1–3 cells**	Ray width 1–3 cells	Ray width 1–3 cells
**Larger rays commonly 4–10-seriate**		
**Aggregate rays**	Aggregate rays	Aggregate rays
**All ray cells procumbent**	All ray cells procumbent	All ray cells procumbent
**Prismatic crystals present**	Prismatic crystals present	Prismatic crystals present
**Prismatic crystals in procumbent ray cells**	Prismatic crystals in procumbent ray cells	Prismatic crystals in procumbent ray cells
**Prismatic crystals in radial alignment in procumbent ray cells**	Prismatic crystals in radial alignment in procumbent ray cells	Prismatic crystals in radial alignment in procumbent ray cells
**Prismatic crystals in chambered axial parenchyma cells**	Prismatic crystals in chambered axial parenchyma cells*	Prismatic crystals in chambered axial parenchyma cells

Supplementary Table 4 | List of fatty acids identified from the residue using LC–MS.

Fatty Acid	Carbons	IUPAC name (PubChem)	RT [minutes]	Chemical Formula
Nonenoic acid	$C_{9:1}$	(E)-non-2-enoic acid	6.94	$C_9H_{16}O_2$
Capric acid	$C_{10:0}$	Decanoic acid	5.82	$C_{10}H_{20}O_2$
2-Decenoic acid	$C_{10:1}$	(E)-dec-2-enoic acid	6.68	$C_{10}H_{18}O_2$
Undecylenic acid	$C_{11:1}$	undec-10-enoic acid	8.69	$C_{11}H_{20}O_2$
Myristoleic acid	$C_{14:1}$	(Z)-tetradec-9-enoic acid	7.67	$C_{14}H_{26}O_2$
Pentadecanoic acid	$C_{15:0}$	Pentadecanoic Acid	5.50	$C_{15}H_{30}O_2$
Palmitic acid	$C_{16:0}$	Hexadecanoic acid	7.90	$C_{16}H_{32}O_2$
Palmitoleic acid	$C_{16:1}$	(Z)-hexadec-9-enoic acid	7.23	$C_{16}H_{30}O_2$
Oleic acid	$C_{18:1}$	(Z)-octadec-9-enoic acid	7.58	$C_{18}H_{34}O_2$
Linoelaidic acid	$C_{18:2}$	(9E,12E)-octadeca-9,12-dienoic acid	8.68	$C_{18}H_{32}O_2$
α-Linolenic acid	$C_{18:3}$	(9Z,12Z,15Z)-octadeca-9,12,15-trienoic acid	7.24	$C_{18}H_{30}O_2$
Linolenelaidic acid	$C_{18:3}$	(9E,12E,15E)-octadeca-9,12,15-trienoic acid	7.62	$C_{18}H_{30}O_2$
Parinaric acid	$C_{18:4}$	(9E,11E,13E,15E)-octadeca-9,11,13,15-tetraenoic acid	7.73	$C_{18}H_{28}O_2$
Arachidonic acid	$C_{20:4}$	(5E,8E,11E,14E)-icosa-5,8,11,14-tetraenoic acid	8.15	$C_{20}H_{32}O_2$
Erucic acid	$C_{22:1}$	(Z)-docos-13-enoic acid	8.67	$C_{22}H_{42}O_2$
Adrenic acid	$C_{22:4}$	(7E,10E,13E,16E)-docosa-7,10,13,16-tetraenoic acid	7.95	$C_{22}H_{36}O_2$

Supplementary References

David, B. et al. Late survival of megafauna refuted for Cloggs Cave, SE Australia: Implications for the Australian Late Pleistocene megafauna extinction debate. *Quat. Sci. Rev.* **253**, 106781, Table 2 (2021). https://doi.org/10.1016/j.quascirev.2020.106781

IAWA Committee. IAWA list of microscopic features for hardwood identification with an Appendix on non-anatomical features. *IAWA Bull.* n.s. **10**(3), 219–332 (1989). https://doi.org/10.1163/22941932-90000496

InsideWood Database. (2004–). http://insidewood.lib.ncsu.edu/search

Moseley M. F. Jr. Comparative anatomy and phylogeny of the Casuarinaceae. *Bot. Gaz.* **110**, 231–280 (1948). https://www.jstor.org/stable/2472487

Wheeler, E. A. Inside Wood—A Web resource for hardwood anatomy. *IAWA J.* **32**(2), 199–211 (2011). https://doi.org/10.1163/22941932-90000051

Wheeler, E. A., Gasson, P. E. & Baas, P. Using the InsideWood Web site: Potentials and pitfalls. *IAWA J.* **41**(4), 412–462 (2020). https://doi.org/10.1163/22941932-bja10032

Wheeler, E. A., Baas, P. & Manchester, S. R. Wood anatomy of modern and fossil Fagales in relation to phylogenetic hypotheses, familial classification, and patterns of character evolution. *Int. J. Plant Sci.* **183**(1), 61–86 (2022). https://doi.org/10.1086/717328
