# Supplementary material for: Archaeological evidence of an ethnographically documented Australian Aboriginal ritual dated to the last ice age
Source: Nat Hum Behav. 2024 Jul 1;8(8):1481–92. doi: 10.1038/s41562-024-01912-w (PMC11343701; doi:10.1038/s41562-024-01912-w)
Supplement: Supplementary file 2 — Reporting Summary [file 41562_2024_1912_MOESM2_ESM.pdf]

nature portfolio

Corresponding author(s): Bruno David

Last updated by author(s): May 4, 2024

Reporting Summary

Nature Portfolio wishes to improve the reproducibility of the work that we publish. This form provides structure for consistency and transparency in reporting. For further information on Nature Portfolio policies, see our Editorial Policies and the Editorial Policy Checklist.

Statistics

For all statistical analyses, confirm that the following items are present in the figure legend, table legend, main text, or Methods section.

n/a	Confirmed	
☒	☐	The exact sample size (*n*) for each experimental group/condition, given as a discrete number and unit of measurement
☒	☐	A statement on whether measurements were taken from distinct samples or whether the same sample was measured repeatedly
☒	☐	The statistical test(s) used AND whether they are one- or two-sided<br>*Only common tests should be described solely by name; describe more complex techniques in the Methods section.*
☒	☐	A description of all covariates tested
☒	☐	A description of any assumptions or corrections, such as tests of normality and adjustment for multiple comparisons
☒	☐	A full description of the statistical parameters including central tendency (e.g. means) or other basic estimates (e.g. regression coefficient) AND variation (e.g. standard deviation) or associated estimates of uncertainty (e.g. confidence intervals)
☒	☐	For null hypothesis testing, the test statistic (e.g. *F*, *t*, *r*) with confidence intervals, effect sizes, degrees of freedom and *P* value noted<br>*Give P values as exact values whenever suitable.*
☒	☐	For Bayesian analysis, information on the choice of priors and Markov chain Monte Carlo settings
☒	☐	For hierarchical and complex designs, identification of the appropriate level for tests and full reporting of outcomes
☒	☐	Estimates of effect sizes (e.g. Cohen's *d*, Pearson's *r*), indicating how they were calculated

Our web collection on statistics for biologists contains articles on many of the points above.

Software and code

Policy information about availability of computer code

Data collection	no software was used
Data analysis	LC-MS data generated in this study were analysed using Xcaliber software and compound identification was performed using the Compound Discoverer v3.2 software with mzCloud, ChemSpider, mzVault and MassList databases. The GC-MS data were analysed using the Chromeleon software.

For manuscripts utilizing custom algorithms or software that are central to the research but not yet described in published literature, software must be made available to editors and reviewers. We strongly encourage code deposition in a community repository (e.g. GitHub). See the Nature Portfolio guidelines for submitting code & software for further information.

Data

Policy information about availability of data

All manuscripts must include a data availability statement. This statement should provide the following information, where applicable:

- Accession codes, unique identifiers, or web links for publicly available datasets
- A description of any restrictions on data availability
- For clinical datasets or third party data, please ensure that the statement adheres to our policy

The authors confirm that all data generated or analysed during this study are included in this published article. The InsideWood Database 2004 (available at http://insidewood.lib.ncsu.edu/search) was used in this study. Additional primary data on excavation depths described in this study are available in references 13 and 19.

The primary data on the two stone artefacts associated with the XU8–9 fireplace are available in reference 15. The primary data on the radiocarbon dates are available in Supplementary Tables 1 and 2. The archaeological materials described in this paper are currently stored in the Monash Indigenous Studies Centre archaeology laboratories and will be returned to the GunaiKurnai Land and Waters Aboriginal Corporation upon completion of the study.

Research involving human participants, their data, or biological material

Policy information about studies with human participants or human data. See also policy information about sex, gender (identity/presentation), and sexual orientation and race, ethnicity and racism.

Reporting on sex and gender	The authors confirm that no research or reporting on sex and gender were undertaken for this study.
Reporting on race, ethnicity, or other socially relevant groupings	The authors confirm that no research or reporting on race, ethnicity or other social group were undertaken for this study.
Population characteristics	The authors confirm that no research or reporting on population characteristics were undertaken for this study.
Recruitment	The authors confirm that no recruiting for research subjects was undertaken for this study.
Ethics oversight	Monash University and the GunaiKurnai Land and Waters Aboriginal Corporation. The ethics protocols were explicitly fully described in a Memorandum of Understanding co-signed on 23 October 2018 by both overseeing institutions.

Note that full information on the approval of the study protocol must also be provided in the manuscript.

Field-specific reporting

Please select the one below that is the best fit for your research. If you are not sure, read the appropriate sections before making your selection.

☐ Life sciences ☒ Behavioural & social sciences ☐ Ecological, evolutionary & environmental sciences

For a reference copy of the document with all sections, see nature.com/documents/nr-reporting-summary-flat.pdf

Behavioural & social sciences study design

All studies must disclose on these points even when the disclosure is negative.

Study description	This is a qualitative archaeological study, reporting on discoveries of specific objects made during an archaeological excavation.
Research sample	The archaeological materials reported here are the entire archaeological collection from the reported levels, rather than samples of larger populations. The archaeological excavation was positioned to determine the antiquity and nature of archaeological materials visible in the exposed sections of an earlier excavation pit.
Sampling strategy	All archaeological materials retained in the 2 mm-mesh sieve, and all those individually collected in situ, were analysed and are reported in the manuscript. All the surfaces of the 2 trimmed wooden artefacts reported in this article were microscopically checked for anthropic modifications.
Data collection	The archaeological excavations were undertaken with small hand trowels and tweezers, and excavated items bagged for laboratory sieving, sorting and analysis. Field notes were made with pen and paper recording. The study did not aim to test any hypothesis, but rather to determine the nature and antiquity of an archaeological discovery. The wood identification specialist in this study was not told about, nor was he aware of, the ethnography until after he had identified the archaeological wood as Casuarina sp. in the lab; he was thus blinded to the independent ethnographic knowledge during the study. The field and laboratory methods are fully reported in the manuscript.
Timing	The archaeological excavations and analysis were undertaken from 5 January 2020 to 19 April 2024.
Data exclusions	No data were excluded from the analysis.
Non-participation	No participants dropped out or declined to participate.
Randomization	All the surfaces of the 2 trimmed wooden artefacts were microscopically examined for anthropic modifications. The residues from the wooden artefact surfaces were sampled from areas after microscopic examination of the surfaces indicated areas of greatest densities. The LC-MS and GC-MS analyses were undertaken on the residues that had been lifted during the original microscopic residue analysis. The archaeology fieldwork rangers were identified by the GunaiKurnai Land and Waters Aboriginal Corporation, representing the Aboriginal Traditional Owners of the study site.

Reporting for specific materials, systems and methods

We require information from authors about some types of materials, experimental systems and methods used in many studies. Here, indicate whether each material, system or method listed is relevant to your study. If you are not sure if a list item applies to your research, read the appropriate section before selecting a response.

Materials & experimental systems

n/a	Involved in the study
☒	☐ Antibodies
☒	☐ Eukaryotic cell lines
☐	☒ Palaeontology and archaeology
☒	☐ Animals and other organisms
☒	☐ Clinical data
☒	☐ Dual use research of concern
☒	☐ Plants

Methods

n/a	Involved in the study
☒	☐ ChIP-seq
☒	☐ Flow cytometry
☒	☐ MRI-based neuroimaging

Palaeontology and Archaeology

Specimen provenance

All the archaeological materials presented in this paper came from Cloggs Cave, East Gippsland (Victoria, Australia), GunaiKurnai Country. The excavations and analyses were undertaken in partnership with the Aboriginal Traditional Owners through the GunaiKurnai Land and Waters Aboriginal Corporation, the Registered Aboriginal Party whose traditional lands includes Cloggs Cave. The research was undertaken under Victorian (Australia) Cultural Heritage Permit GKRAP-19-0001 issued on 14 January 2019 under the Aboriginal Heritage Act 2006.

Specimen deposition

By law in Victoria, Australia, Indigenous cultural materials (including archaeological materials) remain the property of the Aboriginal Traditional Owners. This is the case with all the materials reported here. The samples do not have accession numbers and will be returned to the GunaiKurnai Land and Waters Aboriginal Corporation, who will in due course decide how and where to store or display the materials. No geological or palaeontological specimens were used in our study.

Dating methods

No new radiocarbon dates are presented in this paper. All the dates presented are fully listed and described in Supplementary Information, including their calibrations.

☒ Tick this box to confirm that the raw and calibrated dates are available in the paper or in Supplementary Information.

Ethics oversight

The research was undertaken under a Memorandum of Understanding co-signed by Monash University and the GunaiKurnai Land and Waters Aboriginal Corporation (representing the Aboriginal Traditional Owners of the archaeological site) on 23 October 2018.

Note that full information on the approval of the study protocol must also be provided in the manuscript.

Plants

Seed stocks

n/a

Novel plant genotypes

n/a

Authentication

n/a
